# Overall Survival Benefits of First-Line Treatments for Asian Patients with Advanced Epidermal Growth Factor Receptor-Mutated NSCLC Harboring Exon 19 Deletion: A Systematic Review and Network Meta-Analysis

**DOI:** 10.3390/cancers14143362

**Published:** 2022-07-11

**Authors:** Sik-Kwan Chan, Horace Cheuk-Wai Choi, Victor Ho-Fun Lee

**Affiliations:** 1 Department of Clinical Oncology, School of Clinical Medicine, LKS Faculty of Medicine, The University of Hong Kong, Hong Kong, China; csk01@connect.hku.hk; 2 School of Public Health, LKS Faculty of Medicine, The University of Hong Kong, Hong Kong, China; hcchoi@hku.hk

**Keywords:** non-small-cell lung cancer, epidermal growth factor receptor, exon 19 deletion, tyrosine kinase inhibitors, Asian

## Abstract

**Simple Summary:**

Survival benefits and clinical responsiveness have been exhibited by various generations of EGFR-tyrosine kinase inhibitors (TKIs) in numerous randomized-controlled trials for EGFR-mutated advanced non-small-cell lung cancer (NSCLC) over the past two decades. However, the efficacy, especially long-term overall survival (OS) for Asians harboring an exon 19 deletion (19del) in their NSCLC, remains uncertain. This systematic review and network meta-analysis evaluate the efficacy of all first-line treatments in Asian patients with advanced EGFR-mutated NSCLC harboring 19del. EGFR-TKIs and combination treatments demonstrated no OS benefits in comparison with standard chemotherapy treatments, although progression-free survival (PFS) benefits were revealed. Erlotinib plus bevacizumab, ramucirumab plus erlotinib, and osimertinib are the optimal regimens to prolong PFS for Asians with 19del. Further studies are warranted to investigate the resistance mechanisms and possible strategies for individuals harboring this common mutation.

**Abstract:**

(1) Background: Randomized controlled trials (RCTs) have explored various primary treatments for individuals diagnosed as having later-stage epidermal growth factor receptor (EGFR)-mutated non-small-cell lung cancer. Nevertheless, the extent to which such treatments are efficacious, particularly with regard to overall survival (OS) rates of patients from Asia with exon 19 deletion (19del), has yet to be clarified. (2) Methods: A systematic review and frequentist network meta-analysis were conducted by obtaining pertinent studies from PubMed/MEDLINE Ovid, Embase, Cochrane Library, and trial registries, as well as various other sources. RCTs in which two or multiple treatments in the primary setting for patients from Asia with EGFR 19del were compared were included. This research has been recorded in the Prospective Register of Systematic Reviews (CRD 42022320833). (3) Results: A total of 2715 patients from Asia participated in 18 trials in which 12 different treatments were administered, which included: EGFR tyrosine kinase inhibitors (TKIs) (osimertinib, dacomitinib, afatinib, erlotinib, gefitinib, and icotinib), pemetrexed-based chemotherapy, pemetrexed-free chemotherapy, and combination treatments (gefitinib plus apatinib, erlotinib plus ramucirumab, erlotinib plus bevacizumab, and gefitinib plus pemetrexed-based chemotherapy). Such treatments were not significantly beneficial in terms of OS for patients from Asia who had 19del. It was demonstrated that erlotinib plus bevacizumab, ramucirumab plus erlotinib, and osimertinib consistently yielded the greatest benefits regarding progression-free survival benefit (P-scores = 94%, 84%, and 80%, respectively). Combination treatments resulted in increased toxicity, particularly gefitinib plus apatinib and erlotinib plus bevacizumab, causing the highest prevalence of grade ≥ 3 adverse events. Icotinib and osimertinib had the fewest grade ≥ 3 adverse events. Specific treatments were associated with a wide range of toxicity levels. (4) Conclusions: In patients from Asia with 19del, both EGFR-TKIs and treatments in which therapies were combined exhibited no OS benefits in comparison with standard chemotherapy treatments. Additional research is required to study TKIs’ resistance mechanisms and possible combined approaches for individuals harboring this common mutation.

## 1. Introduction

Lung cancer is the most common cancer worldwide, resulting in approximately 1.8 million fatalities in 2018 [1]. Most of them were reported in Asia, with non-small-cell lung cancer (NSCLC) as the predominant histological type [2,3,4]. Mutation of the epidermal growth factor receptor (EGFR) is more commonly identified in the Asian population (30–40%) than those in the US and Europe (10–15%) [5,6,7,8]. The most common activating mutations among EGFR-mutated NSCLCs are exon 19 deletion (19del) and exon 21 L858R mutation [9,10].

For the past two decades, survival benefits and clinical responsiveness have been exhibited by different generations of EGFR-tyrosine kinase inhibitors (TKIs), which block the cell signaling pathways leading to the proliferation of EGFR-mutated tumors [11]. Thus far, three generations of EGFR-TKIs have been recognized as first-line treatments: erlotinib, gefitinib, and icotinib (first generation); dacomitinib and afatinib (second generation); and osimertinib (third generation) [12]. Further investigated as a simultaneous first-line treatment to extend survival and overcome resistance are the biologically synergistic amalgamations of EGFR-TKIs and monoclonal antibodies, systemic chemotherapy, or other inhibitors of growth pathways, which exhibit various anti-cancer mechanisms [13]. Although a variety of randomized-controlled trials (RCTs) and meta-analyses have reported positive results in the whole study populations [14,15,16,17,18,19,20,21,22,23,24,25,26,27,28,29,30,31,32,33,34,35,36,37,38,39,40], their efficacy remains uncertain, especially with regard to the long-term overall survival (OS) benefit (i.e., the time from treatment to death from any cause) for Asian patients whose tumors harbor EGFR 19del.

In view of the differences in sensitivity and duration of response to EGFR-TKIs as well as survival outcomes, existing studies have suggested that 19del and L858R mutation should be viewed as clinically and biologically distinct entities [41,42,43]. To our knowledge, no meta-analysis or head-to-head investigation has been performed to directly compare the OS benefits of various EGFR-TKIs and their combination treatments in patients with 19del. Following our previous research on the L858R-mutated subgroup [44], we conducted a network meta-analysis (NMA) to evaluate the efficacy of all first-line treatments in Asian patients with advanced EGFR-mutated NSCLC harboring 19del.

## 2. Materials and Methods

### 2.1. Selection Criteria

We performed a systematic literature review of published and unpublished phase II or III RCTs that fulfilled the following inclusion criteria: (1) clinical trials on cytologically or histologically proven advanced (stage III, IV, or recurrent) NSCLC with EGFR mutations; (2) clinical trials comparing two or more arms of first-line treatment for EGFR-mutated NSCLC; (3) clinical trials which Asian patients with EGFR mutations or performed subgroup analyses for Asian patients with EGFR mutations; (4) clinical trials reporting OS, PFS, and toxicity profiles in patients with 19del in their NSCLC. Any clinical trials which reported long-term or mature treatment and survival outcomes regardless of study periods or follow-up durations were eligible for subsequent analysis in this NMA.

### 2.2. Data Sources and Search Strategy

**Figure 1:**
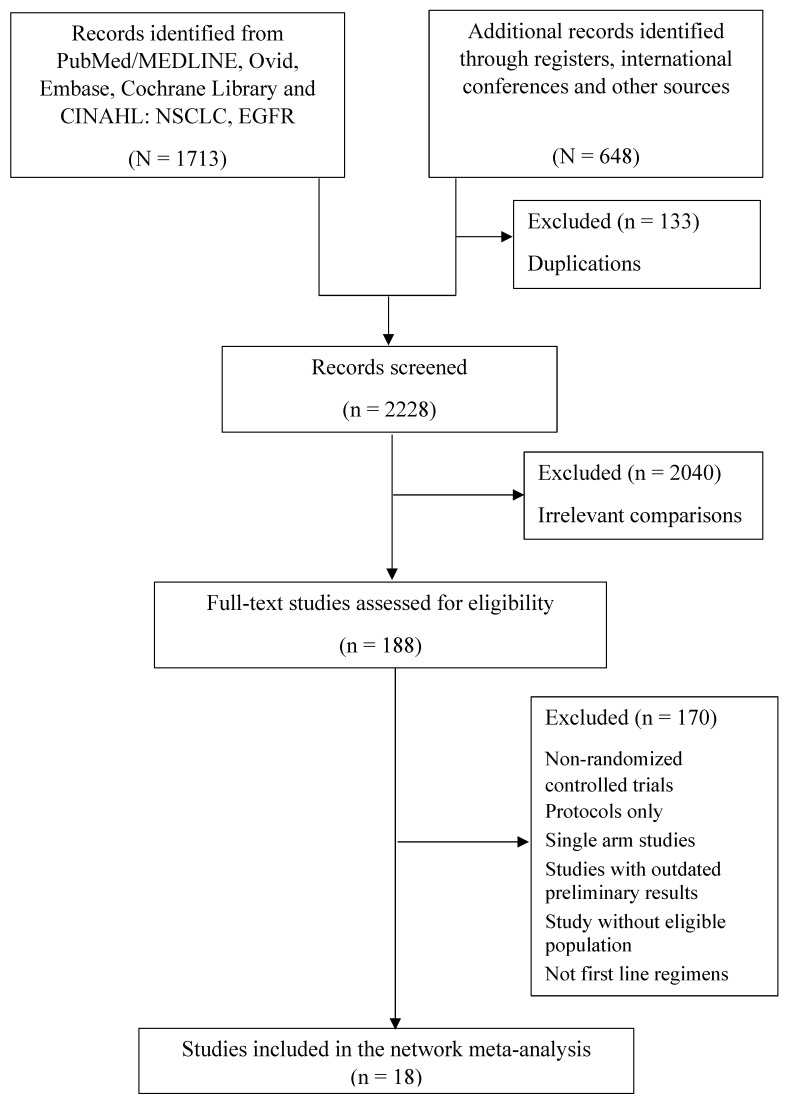
Study flowchart presenting the results of the systematic review.

Our systematic literature search was conducted using PubMed/MEDLINE Ovid, Embase, Cochrane Library, CINAHL Databases, trial registries, and other sources from inception to 30 April 2022, in accordance with the Preferred Reporting Items for Systematic Review and Meta-analysis (PRISMA) guidelines. The search was conducted in all languages using various combinations of the principal search terms “NSCLC” and “EGFR” under the rubric of a “randomised/randomized controlled trial” (Figure 1 and Supplementary Table S1). All titles and abstracts were then screened, following which the full text of all articles eligible for inclusion were reviewed. To ensure the most up-to-date outcomes were included, we also reviewed presentations and abstracts of continuing RCTs on NSCLC from major international conferences (e.g., World Conference on Lung Cancer, European Society of Medical Oncology, and the American Society of Clinical Oncology). To identify additional articles, the reference lists of relevant studies and reviews were manually searched. We restricted this to publications in English only. Full details on the search strategy are available in Supplementary Methods S1 of Supplemental Materials. The procedure employed was registered in the prospective register of systematic reviews (CRD 42022320833).

### 2.3. Data Extraction and Quality Assessment

Two authors (SKC and HCWC) independently extracted the data reported for any applicable variable upon which an analysis was performed. These included the following: (1) study characteristics such as country, year of publication, and phase; (2) number of patients in each arm within the 19del subgroup, treatment protocol, and regimens compared; (3) reported hazard ratio (HR) and 95% confidence interval (CI) for OS and PFS in the 19del subgroup, and (4) incidence of severe AEs (grade ≥ 3) or adverse events (AEs) of any grade as defined in the National Cancer Institute Common Terminology Criteria for AEs. However, because there was no subgroup analysis of AEs, we assumed that the toxicity profile of the 19del subgroup in each trial was similar to that of the overall study population. Although our favored strategy was to extract treatment-related AE, all AEs were included.

Defined as the time from the date of randomization until the date of death from any cause, OS was the primary end point for this NMA. The secondary end points were PFS (the time from the date of randomization to the date of initial disease progression (locoregional or distant) or death from any cause, whichever happened sooner) and AEs of grade ≥ 3.

Two authors (SKC and HCWC) used the Cochrane Collaboration tool to evaluate the risk of bias for each trial according to seven domains connected to biased estimates of the effects of treatment. These were allocation concealment, random sequence generation, blinding of outcome assessment, blinding of participants and personnel, selective reporting, incomplete outcome data, and other sources of bias [45]. Items were rated as having a low, high, or unclear risk of bias. Any disagreements were resolved by the third author (VHFL).

The efficacy of pemetrexed-based chemotherapy (PbCT) was significantly better than that of other chemotherapy regimens in the treatment of non-squamous carcinoma, which is the dominant histological type of EGFR-mutated tumors [46,47,48]. We, therefore, considered PbCT and pemetrexed-free chemotherapy (PfCT) separately in the comparison arms in this NMA. In addition, erlotinib and gefitinib were grouped in the single control arm of the FLAURA Asia trial [49]. Thus, similar to a recently published NMA [48], we assumed these two regimens exhibited identical efficacy in this trial in comparison with the experimental arm of osimertinib.

### 2.4. Statistical Analysis

To compare different treatments in terms of safety and efficacy, all direct and indirect evidence was synthesized and reported as odds ratios (ORs) for binary outcomes (AEs of grade ≥ 3) and HRs for OS and PFS. Their corresponding 95% CIs were also extracted. In view of the superiority in computation and result interpretation, we conducted this NMA under a frequentist framework. The data were analyzed with the R package netmeta (version 4.0.5) [50,51]. To evaluate the heterogeneity among different trials for the same regimen, we used the I^2^ and Q statistics [51]. We also utilized a fixed-effects model and had the option of using a random-effects model in the event of substantial heterogeneity if I^2^ > 50% and/or there was a significant Q statistic (*p* < 0.1). The P-score was used to rank the regimens (the higher the P-score, the better their performance) [52]. To identify whether there was any inconsistency, the NMA results were compared with a standard pairwise meta-analysis. To assess inconsistency for closed loops, a net-splitting analysis was performed to identify significant discrepancies between direct and indirect evidence [53,54,55].

Because there was only a small number of trials in each comparison, we did not generate funnel plots to evaluate publication bias and small study effects. However, to assess the reliability and robustness of the results, we performed a number of sensitivity analyses. In the first sensitivity analysis (further details in Supplementary Methods S2 of Supplementary Materials), we employed a Bayesian approach to reanalyze the data. The second sensitivity analysis was limited to phase III RCTs. The studies of FLAURA Asia and FLAURA China were excluded in the third sensitivity test performed for PFS and grade ≥ 3 AEs for a fairer assessment because the former grouped two regimens in a single control arm, and there were a few overlapping patients in these two studies [48,49,56].

We also evaluated the treatment effects in different contexts in our exploratory analyses. The generation and the reversibility were the treatment nature of interest.

## 3. Results

### 3.1. Systematic Review and Study Characteristics

**Figure 2:**
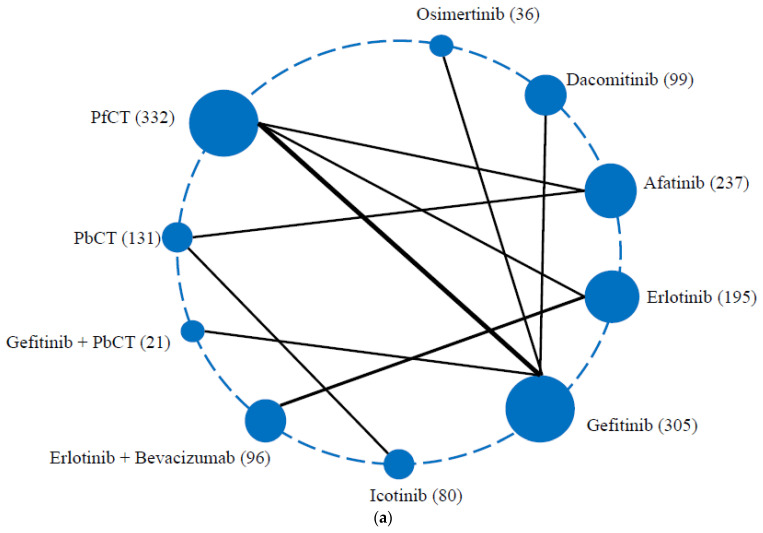

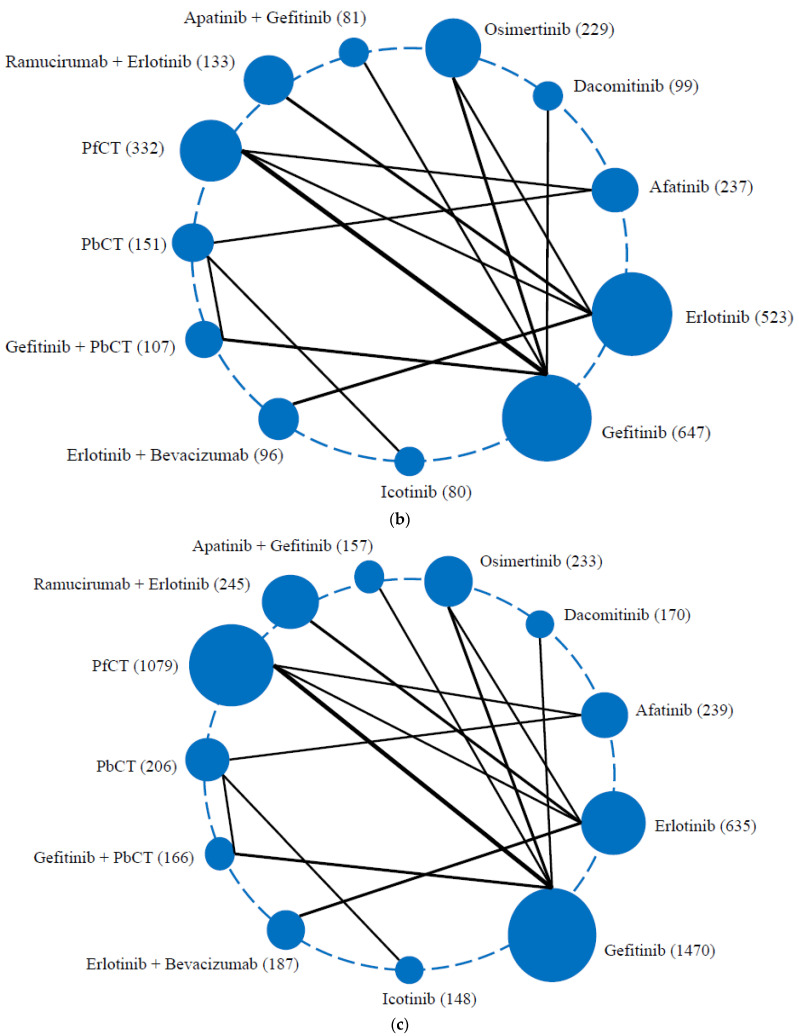
Schematic representation of the network of evidence in this network meta-analysis of first-line treatments in Asian patients with EGFR-mutated NSCLC harboring 19del. (**a**) Overall survival (gefitinib plus apatinib and ramucirumab plus erlotinib were not in this network as the corresponding overall survival data were not reported in trials). (**b**) Progression-free survival. (**c**) Toxicity in terms of grade ≥ 3 adverse events. The network is composed of nodes (treatments) connected by lines (head-to-head comparisons). The size of nodes is proportional to the number of patients in each first-line treatment (in brackets). The width of lines is proportional to the number of comparisons. 19del, exon 19 deletion; EGFR-mutated, epidermal growth factor receptor-mutated; NSCLC, non-small cell lung cancer; PbCT, pemetrexed-based chemotherapy; PfCT, pemetrexed-free chemotherapy.

After initially screening the titles and abstracts of studies, a total of 2361 records was found. The full texts of 188 reports were subsequently accessed and examined (Figure 1). Ultimately, it was determined that 18 reports satisfied the eligibility criteria. Patients who met the criteria in the following studies and rankings (Section 3.1, Section 3.2 and Section 3.3) were included, with treatments, namely EGFR-TKIs (osimertinib, dacomitinib, afatinib, erlotinib, gefitinib, and icotinib), pemetrexed-based chemotherapy, pemetrexed-free chemotherapy, and combination treatments (gefitinib plus apatinib, ramucirumab plus erlotinib, erlotinib plus bevacizumab, and gefitinib plus pemetrexed-based chemotherapy) [17,18,19,20,23,24,26,27,28,29,30,31,32,33,34,36,49,56,57,58,59,60]. Figure 2 depicts the networks. Table 1 presents further details on all the studies that have been included. The risk of potential bias is assessed in Supplementary Figure S1.

### 3.2. Comparison of OS and Ranking

**Figure 3:**
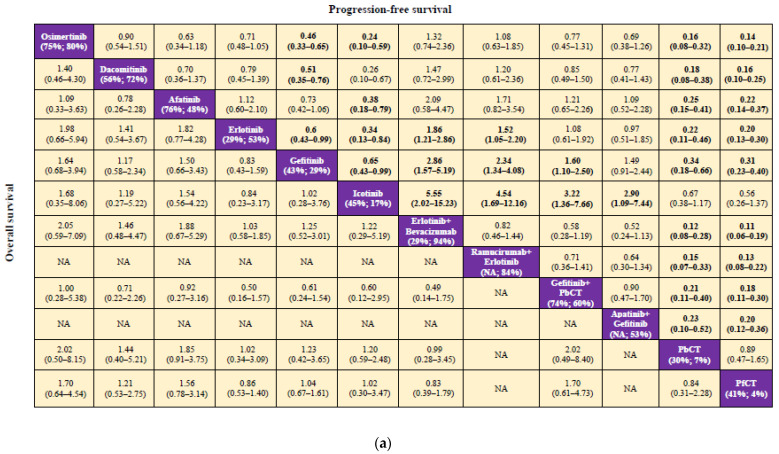

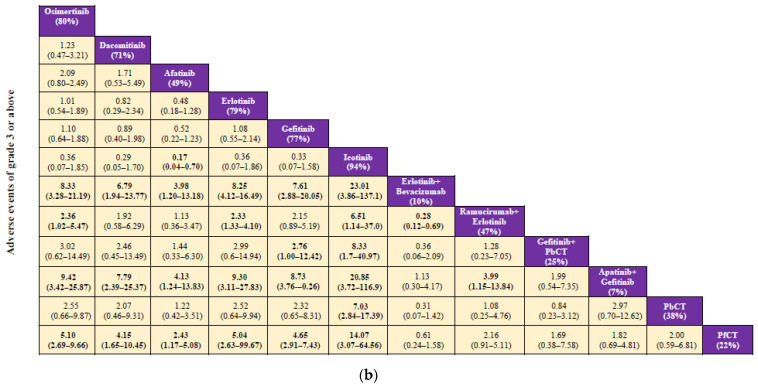
Pooled estimates for the network meta-analysis. (**a**) Pooled hazard ratios (95% confidence intervals) for overall survival (lower triangle) and progression-free survival (upper triangle). P scores for overall survival (left) and progression-free survival (right) are shown beneath the respective treatments. (**b**) Pooled odds ratios (95% confidence intervals) for grade ≥ 3 adverse events. P-scores are indicated below the respective treatments. The data in the cells represent hazard ratios or odds ratios (95% confidence intervals) comparing row-defining treatment versus column-defining treatment. If the value of the hazard ratio or odds ratio is less than 1, then the row-defining treatment is favored. Results considered significant are highlighted in bold. NA, not available; PbCT, pemetrexed-based chemotherapy; PfCT, pemetrexed-free chemotherapy.

The analysis included a total of 13 trials involving 1532 patients from Asia harboring 19del [17,18,19,20,24,26,28,29,31,34,56,57,58]. Significant heterogeneity was not detected (I^2^ = 29%, *p* = 0.398 for Q statistic) and a fixed-effects model was employed. Patients from Asia harboring 19del did not benefit significantly regarding OS from all EGFR-TKIs or combination therapies compared with chemotherapy treatments (Figure 3a and Supplementary Figure S2A). The P-scores for afatinib, osimertinib, and gefitinib plus PbCT were comparatively better with 76%, 75%, and 74%; however, in comparison with various treatments, the differences were not statistically significant.

Exploratory analyses demonstrated that the TKIs from distinct generations were not significantly different with respect to OS with regard to combined therapies and chemotherapy treatments (Supplementary Figure S3A). When ranked in terms of their reversibility, the efficacy levels of the various treatments were largely the same (Supplementary Figure S3B). In comparison with reversible TKIs, irreversible TKIs exhibited potentially greater efficacy (HR 0.67, 95% CI 0.45–0.98).

### 3.3. Comparison of PFS and Ranking

The PFS meta-analysis included a total of 18 studies involving 2715 patients from Asia harboring 19del [17,18,19,20,23,26,27,29,30,31,32,33,36,49,56,58,59,60]. Significant levels of heterogeneity were not detected (I^2^ = 25%, *p* = 0.413 for Q statistic). According to the result of the net-splitting analysis, the direct and indirect estimates were largely consistent (Supplementary Table S2). The majority of the regimens provided considerable PFS benefits in comparison with PfCT (Figure 3a and Supplementary Figure S2B). It was demonstrated that erlotinib plus bevacizumab, ramucirumab plus erlotinib, and osimertinib consistently yielded optimal benefits compared with other regimens regarding PFS, as their P-scores were 94%, 84%, and 80%, respectively.

Exploratory analyses showed that third-generation TKIs (vs. first-generation TKIs (HR 0.53, 95% CI 0.34–0.85) and vs. chemotherapies (0.16, 0.09–0.29)) (P-score = 84%) and first-generation TKI plus antiangiogenic agents (vs. first-generation TKIs (0.62, 0.43–0.88) and vs. chemotherapies (0.19, 0.11–0.30)) (P-score = 78%) provided the highest efficacy in terms of PFS (Supplementary Figure S3A). Moreover, irreversible TKIs and combination treatments were shown to be consistent in yielding the best PFS benefits in patients with 19del (Supplementary Figure S3B).

### 3.4. Safety and Toxicity

Given the reasons aforementioned, the number of patients from the total cohort of 18 studies that were included in this analysis was 4935 [17,18,19,20,23,26,27,29,30,31,32,33,36,49,56,58,59,60]. A fixed-effects model was used as there was no significant heterogeneity (I^2^ = 46.3%, *p* = 0.164 for Q statistic). The net-splitting analysis revealed that the direct and indirect estimates were largely consistent (Supplementary Table S2). In the comparable treatments, fewer toxicities associated with EGFR-TKIs were detected, particularly icotinib and osimertinib, which were linked with the lowest prevalence of grade ≥ 3 AEs, (P-scores = 94% and 80%, respectively) (Figure 3b). In comparison with the different EGFR-TKIs, afatinib was observed to have the highest prevalence of grade ≥ 3 AEs. Additionally, it was found that treatments in which therapies were combined were linked with a greater potential for ≥ 3 AEs (Supplementary Figure S4), where there was an increased likelihood that gefitinib plus apatinib and erlotinib plus bevacizumab would yield the highest grade ≥ 3 AEs (Figure 3b).

From the studies covered by this NMA, over 100 kinds of AEs were reported, of which 16 were chosen because they were considered to be representative of practices implemented in the real world with the greatest clinical relevance [48]. The toxicity profiles of EGFR-TKIs and combination treatments differed from the profiles of traditional chemotherapy treatments as there was an increased prevalence of reports of untoward medical occurrences, including rashes, diarrhea, stomatitis, and interstitial lung disease associated with the former (Supplementary Figure S5).

Moreover, differences were identified in terms of the likelihood that the specified AEs of all grades would occur as a result of the treatments (Supplementary Table S3). Afatinib was linked with the greatest risk of rashes, diarrhea, and stomatitis, with dacomitinib following it in the list. Dacomitinib had the highest likelihood of causing dry skin, interstitial lung disease, and paronychia. Ramucirumab plus erlotinib was linked with an increased risk of liver dysfunction. Osimertinib and erlotinib had relatively mild toxicity profiles, while icotinib had the narrowest and safest one.

### 3.5. Sensitivity Analyses

In the initial sensitivity analysis, the Bayesian approach was employed for the purpose of analyzing OS, PFS, and safety. The results did not indicate any relevant divergence in comparison with the original NMA (Supplementary Figures S6 and S7). Supplementary Table S4 presents a summary of the Bayesian ranking profiles of the treatments investigated. The node splitting analyses did not find significant inconsistency between direct and indirect estimates in the comparisons in PFS and toxicities (Supplementary Table S5). After phase III RCTs were restricted in the subsequent sensitivity analysis, and the remaining treatments in PFS and AEs were compared after the FLAURA studies were removed in the third analysis, the results of the study were confirmed to be robust (Supplementary Figures S8 and S9) [49,56].

## 4. Discussion

To assess the relative effectiveness of first-line treatments for patients with advanced EGFR-mutated NSCLC, multiple scores of RCTs and traditional pairwise meta-analyses were performed [14,15,16,17,18,19,20,21,22,23,24,25,26,27,28,29,30,31,32,33,34,35,36,37,38,39,40]. Unfortunately, these were only based on the direct comparison model and, therefore, did not assess the comparable effectiveness of any two of the numerous first-line treatments. Due to resource limitations and a lengthy event follow-up, it was not possible to carry out a well-designed phase III, multicenter RCT, which makes a direct comparison of all the various first-line treatments. Hence, an NMA is needed to assess the available treatments by generating summary estimates for efficacy between all the various intervention pairs from both direct and indirect comparisons [61,62]. However, earlier NMAs were not specifically applied to Asian patients with 19del and did not include the most up-to-date trials [48,63,64,65,66]. To our knowledge, this NMA is the first to assess different first-line treatments in Asian patients with an advanced EGFR-mutated NSCLC harboring 19del.

In our NMA, we revealed that Asian patients with 19del had no OS benefits with all approved EGFR-TKIs and combination treatment, albeit significant PFS benefits. Besides, significant PFS benefits were demonstrated in combination treatment and irreversible TKIs, with erlotinib plus bevacizumab, erlotinib plus ramucirumab, and osimertinib being the most promising. Furthermore, combination treatment, especially gefitinib plus apatinib and erlotinib plus bevacizumab, resulted in more toxicities, while icotinib and osimertinib produced the fewest grade ≥ 3 adverse events. EGFR-TKIs were associated with different toxicity spectrums. Finally, sensitivity analyses demonstrated the robustness of our study results.

In line with a previous study, no significant efficacy of EGFR-TKIs or combination therapies in terms of OS was demonstrated for the Asian 19del subgroup in our NMA following the inclusion of the up-to-date clinical trials [39,49,56,59,60]. The performance in PFS and safety of the treatments assessed in the current NMA were in compliance with those in the previously published pooled analysis and NMA. Our recently published NMA for L858R mutation revealed that gefitinib plus PbCT was the most efficacious in prolonging PFS, while the current NMA for 19del indicated that erlotinib plus bevacizumab or ramucirumab were the most optimal one and this further demonstrated the potential difference in tumor biology and sensitivity to EGFR-TKIs between these two mutant subgroups [44,48]. Possible explanations include that simultaneous treatment with gefitinib and PbCT was previously reported to delay the resistance to gefitinib mediated by T790M mutation, which is more associated with L858R mutation, while combining EGFR-TKIs and antiangiogenic agents with the dual blockade of the EGFR and VEGF pathways alters the T790M resistance mechanism pathway in a very limited way [59,60,67]. Combination treatments, however, imply additional adverse events for either combined drug. Following multiple comparisons in this NMA, erlotinib plus bevacizumab is one of the regimens resulting in the worst safety profile. Clinicians should keep in mind the toxicity profile of each regimen in particular when prescribing combination treatments. While we tried our best to summarize the major acute toxicities in this study with an assumption that the toxicity profiles of the 19del subgroup were comparable with those in the overall study population, additional studies are warranted for a comprehensive comparison of the toxicity spectrum of each treatment specifically for Asians with 19del.

Our study provides crucial evidence for clinicians to evaluate different EGFR-TKI treatment options for Asian patients with EGFR 19del NSCLC. Nonetheless, there are several issues needed to be further addressed in the future. First of all, understanding the mechanism of resistance to EGFR-TKIs in the 19del subgroup in further studies may help delay resistance and provide therapeutic benefits, especially long-term OS efficacy. MET amplification, which is more associated with 19del, is one of the most common mechanisms of resistance following treatment with all generations of EGFR-TKIs, especially osimertinib [68,69]. Furthermore, molecular subtypes of 19del showed differences in TKI sensitivity. Preclinical studies revealed that L747-A750 > P variant shows inferior outcomes when treated with erlotinib or osimertinib, while it retains high sensitivity to afatinib and dacomitinib [70,71]. C-helix 19del (i.e., deletion occurring in the C-helix part of exon 19) being differentiated from the classical 19del may also be a potential predictor of clinical outcomes following treatment with EGFR-TKIs [72]. Several retrospective studies, including ours, revealed that different molecular subtypes of 19del with or without additional insertional mutations conferred variable survival outcomes and responses to different generations of TKIs [71,72,73,74,75]. These underline the importance of incorporating comprehensive genomic profiling with next-generation sequencing into initial treatment planning to detect such variations for a more personalized treatment strategy [76,77]. In addition, the demonstrated efficacy of the current combination strategy provides insights into the exploration of the combination of osimertinib and other regimens. Trials such as FLAURA2 (NCT04035486) of combining osimertinib and chemotherapy, RAMOSE (NCT03909334) and TORG1833 (JPRN-JapicCTI-184146) evaluating osimertinib plus ramucirumab are ongoing [78,79,80]. It is also interesting to await the results of SAVANNAH (NCT03778229), combining osimertinib and a MET inhibitor [81]. Other clinical trials testing the combination of other EGFR-TKIs and a MET inhibitor, including MARQUEE (NCT01244191), INSIGHT (NCT01982955), MetLung (NCT01456325), and NCT01610336, may also provide some clues [82,83,84,85]. Overall, 19del should be regarded as a distinct group, while the current international guidelines grouping 19del and L858R mutation into a single category and treating them by the same strategy deviate from the principles of precision medicine [12]. Further studies are warranted to investigate the most optimal treatment strategy for Asian NSCLC patients with different mutation subgroups.

Our NMA has a number of strengths in comparison with other reported NMAs and meta-analyses for patients with advanced EGFR-mutated NSCLC [36,37,38,39,40,48,63,64,65,66]. Firstly, it is the most up-to-date study to compare all current EGFR-TKI monotherapies with other combination treatments and systemic chemotherapy as a first-line treatment for Asian patients with 19del. Secondly, we conducted a comprehensive analysis of all primary indicators of effectiveness, including OS and PFS and toxicity outcomes, utilizing a meticulous methodology and a wide-ranging and up-to-date body of data which includes both recently updated and previously unpublished results. We are eagerly awaiting the mature OS data of CTONG1706 and RELAY [36,59,60]. Furthermore, in addition to the aforementioned FLAURA2, RAMOSE and TORG1833, and MARIPOSA (NCT04487080), examining the combination of lazertinib and amivantamab, are also underway [78,79,80,85].

However, there are also some limitations in our work that need to be addressed. Firstly, even though a variety of public health bodies now recognize NMA as a valid way to assess healthcare interventions [61,62], there are a number of inevitable drawbacks that arise from its use of indirect comparisons [55,86,87]. For instance, even though it was useful to rank regimens in terms of OS, PFS, and grade ≥ 3 AEs, these were principally calculated using point estimates, namely HRs and ORs [52]. To evaluate the evidence and the superior performance of a particular regimen precisely and critically, there should be a greater focus on HR or OR estimates and their respective CIs, including the extent to which they are consistent across a range of end-points. Secondly, the estimates may have been potentially less precise as a result of the inclusion of FLAURA studies that grouped erlotinib and gefitinib together in the same control arm [49,56]. Nevertheless, to ensure robust results, sensitivity analyses were performed, which excluded FLAURA studies. Thirdly, the lack of specific information in the 19del subgroup clarifying whether the major end point, OS, was confounded by the subsequent treatment lines and the nature of crossing-over to the experimental regimen is an unresolved issue. Besides, in some trials, the mature OS in their interim analysis was not reported, or survival data for Asian patients with 19del were lacking. However, to provide a more comprehensive review of the efficacy of treatment, we reported PFS as the secondary outcome measure. Furthermore, our NMA cannot provide further information on the differential survival outcomes of different first-line treatments among the various molecular subtypes of 19del, which were not available in the included RCTs. Finally, it is difficult to ascertain whether optimal randomization and balancing of patients within the 19del subgroup occurred in the RCTs included in our NMA. Moreover, the lack of descriptive statistics such as age, sex, and performance status for the baselines in this subgroup meant that it was not possible to assess transitivity (i.e., similarity between trials in terms of study characteristics). Future work, such as an individual patient data NMA, should assess the comparable treatment effectiveness in the 19del subgroup more precisely, which the current NMA could not achieve as relevant data were missing from current studies.

## 5. Conclusions

To conclude, Asian patients with 19del demonstrated no OS benefit with all approved EGFR-TKIs and combination treatments despite significant PFS benefits. Clinical judgment should be carefully exercised with the evaluation of the treatment toxicities in a comprehensive way. Follow-up trial data and further clinical studies are warranted for the sake of a more personalized treatment strategy for this mutation subgroup.

## Figures and Tables

**Table 1:** Baseline characteristics of studies included in the network meta-analysis.

Study	Phase	Sample Size (No.)	Intervention Arm	Control Arm	Reported OS (HR, 95% CI)	Reported PFS (HR, 95% CI)
NEJ026 [30,57]	III	56/55	Erlotinib 150 mg once a day + bevacizumab 15 mg/kg every 3 weeks	Erlotinib 150 mg once a day	1.34 (0.76–2.37)	0.69 (0.41–1.16)
FLAURA Asia [49]	III	193	Osimertinib 80 mg once a day	Gefitinib 250 mg once a day or Erlotinib 150 mg once a day	NR	0.59 (0.41–0.85)
FLAURA China [56]	III	36/33	Osimertinib 80 mg once a day	Gefitinib 250 mg once a day	0.61 (0.32–1.18)	0.41 (0.22–0.77)
ARCHER Asia [58]	III	99/103	Dacomitinib 45 mg once a day	Gefitinib 250 mg once a day	0.86 (0.59–1.24)	0.51 (0.36–0.61)
COVINCE [17]	III	80/74	Icotinib 125 mg three times a day	PbCT (cisplatin 75 mg/m^2^ + pemetrexed 500 mg/m^2^ every 3 weeks (4 cycles) + pemetrexed 500 mg/m^2^ every 3 weeks)	0.83 (0.55–1.27)	0.67 (0.42–1.09)
Han et al. [31]	II	21/21	Gefitinib 250 mg once a day + PbCT (carboplatin AUC = 5 + pemetrexed 500 mg/m^2^ every 4 weeks (6 cycles) + pemetrexed 500 mg/m^2^ every 4 weeks)	Gefitinib 250 mg once a day	0.61 (0.30–1.25)	0.60 (0.30–1.21)
		21/20	Gefitinib 250 mg once a day + PbCT (carboplatin AUC = 5 + pemetrexed 500 mg/m^2^ every 4 weeks (6 cycles) + pemetrexed 500 mg/m^2^ every 4 weeks)	PbCT (carboplatin AUC = 5 + pemetrexed 500 mg/m2 every 4 weeks (6 cycles) + pemetrexed 500 mg/m^2^ every 4 weeks)	NR	0.15 (0.06–0.36)
JMIT [32]	II	65/40	Gefitinib 250 mg once a day + pemetrexed 500 mg/m^2^ every 3 weeks	Gefitinib 250 mg once a day	NR	0.67 (0.43–1.05)
ENSURE [18]	III	57/61	Erlotinib 150 mg once a day	PfCT (gemcitabine 1250 mg/m^2^ + cisplatin 75 mg/m^2^ every 3 weeks (≤4 cycles))	0.79 (0.48–1.30)	0.20 (0.11–0.37)
JO25567 [33,34]	II	40/40	Erlotinib 150 mg once a day + bevacizumab 15 mg/kg every 3 weeks	Erlotinib 150 mg once a day	0.79 (0.44–1.44)	0.41 (0.24–0.72)
LUX-Lung 6 [20]	III	124/62	Afatinib 40 mg once a day	PfCT (gemcitabine 1000 mg/m^2^ + cisplatin 75 mg/m^2^ every 3 weeks (≤6 cycles))	0.64 (0.44–0.94)	0.20 (0.13–0.33)
LUX-Lung 3 [19]	III	113/57	Afatinib 40 mg once a day	PbCT (cisplatin 75 mg/m^2^ + pemetrexed 500 mg/m^2^ every 3 weeks (≤6 cycles))	0.34 (0.13–0.87)	0.16 (0.06–0.39)
OPTIMAL [23,24]	III	43/39	Erlotinib 150 mg once a day	PfCT (gemcitabine 1000 mg/m^2^ + cisplatin AUC = 5 every 3 weeks (≤4 cycles))	1.52 (0.92–2.52)	0.13 (0.07–0.25)
NEJ002 [26]	III	58/59	Gefitinib 250 mg once a day	PfCT (paclitaxel 200 mg/m^2^ + carboplatin AUC = 6 every 3 weeks (≥3 cycles))	0.83 (0.52–1.54)	0.24 (0.15–0.38)
WJTOG [27,28]	III	50/37	Gefitinib 250 mg once a day	PfCT (cisplatin 80 mg/m^2^ + docetaxel 60 mg/m^2^ every 3 weeks (3–6 cycles))	1.41 (0.85–2.34)	0.45 (0.27–0.77)
RELAY (East Asian) [59]	III	84/84	Ramucirumab 10 mg/kg every 2 weeks + Erlotinib 150 mg once a day	Erlotinib 150 mg once a day	NR	0.63 (0.43–0.92)
RELAY (Japanese) [60]	III	49/51	Ramucirumab 10 mg/kg every 2 weeks + Erlotinib 150 mg once a day	Erlotinib 150 mg once a day	NR	0.70 (0.42–1.16)
IPASS [29]	III	66/74	Gefitinib 250 mg once a day	PfCT (paclitaxel 200 mg/m^2^ + carboplatin AUC = 5/6 every 3 weeks (3–6 cycles))	0.79 (0.54–1.15)	0.38 (0.26–0.56)
CTONG1706 [36]	III	81/83	Apatinib 500 mg + Gefitinib 250 mg once a day	Gefitinib 250 mg once a day	NR	0.67 (0.45–0.99)

AUC, area under the concentration-time curve; HR, hazard ratio; NR, not reported; OS, overall survival; PbCT, pemetrexed-based chemotherapy; PfCT, pemetrexed free chemotherapy; PFS, progression-free survival.

## Data Availability

All articles in this manuscript are available from MEDLINE, Embase, Cochrane Library, CINAHL, and Google Scholar.

## Supplementary Materials

The following supporting information can be downloaded at: https://www.mdpi.com/article/10.3390/cancers14143362/s1; Methods S1: Literature Search Criteria; Methods S2: Methods of Bayesian Approach; Table S1: PRISMA Checklist for the Network Meta-analysis; Table S2: Frequentist Net-splitting Analysis of Inconsistency; Table S3: (A) Relative toxicity (pooled odds ratios and 95% confidence interval) of treatments on seven commonly reported specific adverse events (any grade) based on the overall population of each treatment included in the network meta-analysis. (B) P-score of each comparable treatment for each specific adverse event (any grade); Table S4: Bayesian Ranking Results of Network Meta-analysis in the First Sensitivity Analysis; Table S5: Bayesian Node-splitting Analysis of Inconsistency in the First Sensitivity Analysis; Figure S1: (A) Summary of the risk of bias assessment in 7 domains in the 18 studies included in the network meta-analysis. (B) Graphical representation of the overall risk of bias in the 7 domains; Figure S2: Forest plot for (A) overall survival and (B) progression-free survival showing results comparing treatment regimens against pemetrexed-free chemotherapy from the network meta-analysis; Figure S3: Pooled estimates of the exploratory analyses for overall survival and progression-free survival. (A) Pooled hazard ratios (95% confidence intervals) for overall survival and progression-free survival stratified by the generation of treatment regimens. (B) Pooled hazard ratios (95% confidence intervals) for overall survival and progression-free survival stratified by the reversibility of the treatment regimens; Figure S4: Pooled estimates of the exploratory analyses for adverse events of grade ≥3; Figure S5: Toxicity profile in relation to the incidence (%) of each specific adverse event (any grade) based on the overall population of each treatment included in the network meta-analysis; Figure S6: Pooled estimates of the network meta-analysis in the first sensitivity analysis by Bayesian approach; Figure S7: Convergence of the three chains established by inspection of the history feature and the Brooks–Gelman–Rubin diagnostic for (A) overall survival, (B) progression-free survival, and (C) adverse events of grade ≥ 3; Figure S8: Pooled estimates of the network meta-analysis after including only phase III trials in the second sensitivity analysis; Figure S9: Pooled estimates of the network meta-analysis after excluding FLAURA studies in the third sensitivity analysis.
