# Supplementary material for: Overall Survival Benefits of First-Line Treatments for Asian Patients with Advanced Epidermal Growth Factor Receptor-Mutated NSCLC Harboring Exon 19 Deletion: A Systematic Review and Network Meta-Analysis
Source: Cancers (Basel). 2022 Jul 11;14(14):3362. doi: 10.3390/cancers14143362 (PMC9316403; doi:10.3390/cancers14143362)
Supplement: Supplementary file 1 [file cancers-14-03362-s001.zip › cancers-1801634-supplementary.pdf]

Overall Survival Benefits of First-Line Treatments for Asian Patients with Advanced Epidermal Growth Factor Receptor-Mutated NSCLC Harboring Exon 19 Deletion: A Systematic Review and Network Meta-Analysis

Sik-Kwan Chan, Horace Cheuk-Wai Choi and Victor Ho-Fun Lee

Methods S1. Literature Search Criteria.

((((((((((((non-small-cell lung cancer[title] OR non-small cell lung cancer[title]) OR non small-cell lung cancer[title]) OR non small cell lung cancer[title]) OR non-small-cell lung carcinoma[title]) OR non-small cell lung carcinoma[title]) OR non small-cell lung carcinoma[title]) OR non small cell lung carcinoma[title]) OR nsclc[title]) AND (epidermal growth factor receptor[title/abstract] OR EGFR[title/abstract])) AND (((((((((((((((treatment[title/abstract] OR therapy[title/abstract]) OR tyrosine kinase inhibitor[title/abstract]) OR TKI[title/abstract]) OR osimertinib[title/abstract]) OR dacomitinib[title/abstract]) OR afatinib[title/abstract]) OR erlotinib[title/abstract]) OR gefitinib[title/abstract]) OR icotinib[title/abstract]) OR chemotherapy[title/abstract]) OR first-line[title/abstract]) OR first line[title/abstract]) OR treatment-naive[title/abstract]) OR treatment-naïve[title/abstract]) OR untreated[title/abstract])) AND (((((compare[title/abstract] OR comparison[title/abstract]) OR comparative[title/abstract]) OR comparing[title/abstract]) OR versus[title/abstract]) OR vs[title/abstract])) AND ((((((Randomized Controlled Trial[ptyp] OR controlled clinical trial[ptyp]) OR randomized[title/abstract]) OR randomised[title/abstract]) OR randomly[title/abstract]) OR trial[title/abstract]) OR phase[title/abstract])) AND ("0001/01/01"[Date - Publication] : "2022/04/30"[Date - Publication])

Methods S2. Methods of Bayesian Approach.

Network meta-analysis (NMA) within the Bayesian framework in the first sensitivity analysis was performed using Markov Chain Monte Carlo simulation technique with the R package gemtc (version 4.0.5, R Foundation for Statistical Computing, Vienna, Austria). For each outcome measure, a fixed-effects consistency model was used, as most direct evidence was from one trial. Three independent Markov chains were established for running 100,000 burn-ins and 150,000 sample iterations per chain simultaneously with a thinning interval of 1. This process was intended to obtain a posterior distribution, with model convergence of the iterations being evaluated and visualized using Brooks-Gelman-Rubin diagnostic (Supplementary Figure. 7). Once convergence was established, the posterior distributions for the model parameters were obtained as the output of the NMA estimate (hazard/odds ratio and the corresponding 95% credible interval). In the presence of minimally informative priors, credible intervals can be interpreted like conventional confidence intervals.[2] The Bayesian approach also provided overall ranking probabilities for each regimen, making it possible to rank each outcome measurement from the best to the worst, and were then visualized by calculating the surface under the cumulative ranking curves on the basis of the ranking profiles [87]. The node splitting approach was used to calculate the inconsistency of the model for closed loops in the network where direct and indirect evidence were separately contrasted on a particular comparison (node) [53, 54, 88]. Significant inconsistency was indicated if node-splitting analysis derived $P < 0.05$ of disagreement between direct and indirect evidence.

Table S1. PRISMA checklist for the network meta-analysis.

Section and Topic	Item #	Checklist item	Location where item is reported
**TITLE**			
Title	1	Identify the report as a systematic review.	1, 3
**ABSTRACT**			
Abstract	2	See the PRISMA 2020 for Abstracts checklist.	3–4
**INTRODUCTION**			
Rationale	3	Describe the rationale for the review in the context of existing knowledge.	5–6
Objectives	4	Provide an explicit statement of the objective(s) or question(s) the review addresses.	5–6
**METHODS**			
Eligibility criteria	5	Specify the inclusion and exclusion criteria for the review and how studies were grouped for the syntheses.	6
Information sources	6	Specify all databases, registers, websites, organisations, reference lists and other sources searched or consulted to identify studies. Specify the date when each source was last searched or consulted.	6–7
Search strategy	7	Present the full search strategies for all databases, registers and websites, including any filters and limits used.	6–7
Selection process	8	Specify the methods used to decide whether a study met the inclusion criteria of the review, including how many reviewers screened each record and each report retrieved, whether they worked independently, and if applicable, details of automation tools used in the process.	7–8
Data collection process	9	Specify the methods used to collect data from reports, including how many reviewers collected data from each report, whether they worked independently, any processes for obtaining or confirming data from study investigators, and if applicable, details of automation tools used in the process.	7–8
Data items	10a	List and define all outcomes for which data were sought. Specify whether all results that were compatible with each outcome domain in each study were sought (e.g. for all measures, time points, analyses), and if not, the methods used to decide which results to collect.	7–8
	10b	List and define all other variables for which data were sought (e.g. participant and intervention characteristics, funding sources). Describe any assumptions made about any missing or unclear information.	8
Study risk of bias assessment	11	Specify the methods used to assess risk of bias in the included studies, including details of the tool(s) used, how many reviewers assessed each study and whether they worked independently, and if applicable, details of automation tools used in the process.	8–9
Effect measures	12	Specify for each outcome the effect measure(s) (e.g. risk ratio, mean difference) used in the synthesis or presentation of results.	8–9
Synthesis methods	13a	Describe the processes used to decide which studies were eligible for each synthesis (e.g. tabulating the study intervention characteristics and comparing against the planned groups for each synthesis (item #5)).	8–9
	13b	Describe any methods required to prepare the data for presentation or synthesis, such as handling of missing summary statistics, or data conversions.	8–9
	13c	Describe any methods used to tabulate or visually display results of individual studies and syntheses.	8–9
	13d	Describe any methods used to synthesize results and provide a rationale for the choice(s). If meta-analysis was performed, describe the model(s), method(s) to identify the presence and extent of statistical heterogeneity, and software package(s) used.	8–9
	13e	Describe any methods used to explore possible causes of heterogeneity among study results (e.g. subgroup analysis, meta-regression).	8–9
	13f	Describe any sensitivity analyses conducted to assess robustness of the synthesized results.	8–9
Reporting bias assessment	14	Describe any methods used to assess risk of bias due to missing results in a synthesis (arising from reporting biases).	8–9

Section and Topic	Item #	Checklist item	Location where item is reported
Certainty assessment	15	Describe any methods used to assess certainty (or confidence) in the body of evidence for an outcome.	8–9
**RESULTS**			
Study selection	16a	Describe the results of the search and selection process, from the number of records identified in the search to the number of studies included in the review, ideally using a flow diagram.	10
	16b	Cite studies that might appear to meet the inclusion criteria, but which were excluded, and explain why they were excluded.	10
Study characteristics	17	Cite each included study and present its characteristics.	10
Risk of bias in studies	18	Present assessments of risk of bias for each included study.	10
Results of individual studies	19	For all outcomes, present, for each study: (a) summary statistics for each group (where appropriate) and (b) an effect estimate and its precision (e.g. confidence/credible interval), ideally using structured tables or plots.	10–12
Results of syntheses	20a	For each synthesis, briefly summarise the characteristics and risk of bias among contributing studies.	10–12
	20b	Present results of all statistical syntheses conducted. If meta-analysis was done, present for each the summary estimate and its precision (e.g. confidence/credible interval) and measures of statistical heterogeneity. If comparing groups, describe the direction of the effect.	10–13
	20c	Present results of all investigations of possible causes of heterogeneity among study results.	10–13
	20d	Present results of all sensitivity analyses conducted to assess the robustness of the synthesized results.	12–13
Reporting biases	21	Present assessments of risk of bias due to missing results (arising from reporting biases) for each synthesis assessed.	10
Certainty of evidence	22	Present assessments of certainty (or confidence) in the body of evidence for each outcome assessed.	10–13
**DISCUSSION**			
Discussion	23a	Provide a general interpretation of the results in the context of other evidence.	13–14
	23b	Discuss any limitations of the evidence included in the review.	15–16
	23c	Discuss any limitations of the review processes used.	15–16
	23d	Discuss implications of the results for practice, policy, and future research.	15–16
**OTHER INFORMATION**			
Registration and protocol	24a	Provide registration information for the review, including register name and registration number, or state that the review was not registered.	7
	24b	Indicate where the review protocol can be accessed, or state that a protocol was not prepared.	7
	24c	Describe and explain any amendments to information provided at registration or in the protocol.	7
Support	25	Describe sources of financial or non-financial support for the review, and the role of the funders or sponsors in the review.	NA
Competing interests	26	Declare any competing interests of review authors.	NA
Availability of data, code and other materials	27	Report which of the following are publicly available and where they can be found: template data collection forms; data extracted from included studies; data used for all analyses; analytic code; any other materials used in the review.	NA

PRISMA, Preferred Reporting Items for Systematic Reviews and Meta-Analysis.

Table S2. Frequentist net-splitting analysis of inconsistency.

Nets	Direct Effect	Indirect Effect	Overall	*P*
**Progression-free survival**				
Afatinib, PbCT	0.28 (0.16–0.58)	0.15 (0.09–0.28)	0.25 (0.15–0.41)	0.341
Afatinib, PfCT	0.20 (0.09–0.42)	0.38 (0.18–0.55)	0.22 (0.14–0.37)	0.342
Erlotinib, Osimertinib	1.69 (0.96–2.34)	0.94 (0.88–1.66)	1.41 (0.93–2.08)	0.162
Erlotinib, PfCT	0.16 (0.08–0.28)	0.29 (0.14–0.35)	0.20 (0.13–0.30)	0.158
Gefitinib, Gefitinib+PbCT	1.55 (1.03–2.16)	2.94 (1.73–3.62)	1.60 (1.10–2.50)	0.342
Gefitinib, Osimertinib	1.90 (1.21–2.65)	3.44 (1.82–4.31)	2.17 (1.57–3.03)	0.162
Gefitinib, PfCT	0.34 (0.21–0.49)	0.18 (0.12–0.24)	0.31 (0.23–0.40)	0.081
Gefitinib+PbCT, PbCT	0.15 (0.09–0.26)	0.28 (0.17–0.52)	0.21 (0.11–0.40)	0.341
**Adverse events of grade ≥3**				
Afatinib, PbCT	1.03 (0.39–2.74)	0.22 (0.11–0.84)	0.82 (0.28–2.38)	0.311
Afatinib, PfCT	0.37 (0.23–0.58)	1.36 (0.42–1.58)	0.42 (0.19–0.85)	0.321
Erlotinib, Osimertinib	1.38 (0.89–1.17)	0.79 (0.55–1.23)	1.01 (0.54–1.89)	0.138
Erlotinib, PfCT	0.19 (0.06–0.52)	0.14 (0.03–0.28)	0.20 (0.01–0.38)	0.178
Gefitinib, Gefitinib+PbCT	0.16 (0.07–0.48)	0.77 (0.24–1.52)	0.36 (0.08–1.00)	0.311
Gefitinib, Osimertinib	0.89 (0.64–3.21)	2.50 (1.23–3.10)	1.10 (0.53–1.85)	0.138
Gefitinib, PfCT	0.26 (0.18–0.42)	0.09 (0.04–0.24)	0.21 (0.13–0.34)	0.102
Gefitinib+PbCT, PbCT	0.80 (0.20–3.22)	3.79 (1.44–4.86)	1.19 (0.32–4.34)	0.312

* The frequentist net-splitting analysis was applied to evaluate inconsistency for closed loops in the network. No closed loop is found in the network of overall survival. PbCT, pemetrexed-based chemotherapy; PfCT, pemetrexed-free chemotherapy.

Table S3. (**A**) Relative toxicity (pooled odds ratios and 95% confidence interval) of treatments on seven commonly reported specific adverse events (any grade) based on the overall population of each treatment included in the network meta-analysis. Significant values are in bold and colored in gray (less toxicity) and light yellow (more toxicity). (**B**) P-score of each comparable treatment for each specific adverse event (any grade). Lower P-scores indicate higher probability to cause each specific adverse event. Treatments with the highest risk of each adverse event are colored in red.

A

Comparison	Pooled odds ratio (95% confidence interval)						
	**Rash**	**Diarrhea**	**Stomatitis**	**Paronychia**	**Dry skin**	**Liver dysfunction#**	**ILD**
				**versus Osimertinib**			
Dacomitinib	0.90 (0.15–5.55)	**6.78 (2.08–22.10)**	2.18 (0.95–5.02)	1.68 (0.29–9.69)	2.22 (0.50–9.94)	3.45 (0.89–13.4)	1.40 (0.20–9.60)
Afatinib	5.14 (0.98–26.8)	**36.22 (12.40–105.90)**	**4.33 (1.80–10.40)**	1.64 (0.26–10.33)	0.86 (0.41–2.52)	3.56 (0.78–16.2)	0.20 (0.02–1.67)
Erlotinib	2.77 (0.87–8.85）	1.47 (0.69–3.13)	0.88 (0.52–1.51)	0.34 (0.11–1.03)	0.6 (0.21–1.73)	**8.98 (2.32–34.80)**	0.18 (0.05–0.68)
Gefitinib	1.96 (0.75–5.14)	0.93 (0.50–1.73)	0.54 (0.34–0.83)	0.63 (0.25–1.59)	1.56 (0.69–3.55)	**9.10 (3.48–23.70)**	0.80 (0.28–2.28)
Icotinib	0.32 (0.03–3.26)	0.36 (0.08–1.61)	NA	NA	NA	3.13 (0.52–18.70)	0.09 (0.01–2.01)
Erlotinib+ Bevacizumab	2.83 (0.57–13.90)	1.85 (0.66–5.20)	1.39 (0.67–2.90)	0.43 (0.09–1.99)	NA	**6.82 (1.50–31.00)**	0.05 (0.01–0.26)
Ramucirumab+ Erlotinib	NA	1.85 (0.66–5.20)	1.73 (0.83–3.59)	NA	0.66 (0.13–3.40)	**20.30 (4.40–92.30)**	0.07 (0.01–0.43)
Gefitinib+ PbCT	1.08 (0.21–5.54)	0.81 (0.33–2.00)	0.99 (0.43–2.28)	NA	0.97 (0.22–4.34)	**14.50 (4.80–46.80)**	0.82 (0.12–5.71)
Apatinib +Gefitinib	1.86 (0.30–11.50)	NA	1.36 (0.59–3.12)	NA	NA	**13.60 (3.50–52.50)**	NA
PbCT	**0.03 (0.01–0.15)**	**0.16 (0.05–0.48)**	**0.15 (0.05–0.45)**	**0.01 (0.00–0.03)**	**0.04 (0.02–0.08)**	**4.81 (1.10–21.80)**	**0.1 (0.01–1.38)**
PfCT	**0.17 (0.06–0.52)**	**0.25 (0.12–0.52)**	**0.23 (0.13–0.38)**	**0.02 (0.01–0.04)**	**0.07 (0.02–0.24)**	2.9 (0.92–9.68)	**0.22 (0.66–0.81)**
				**versus Dacomitinib**			
Afatinib	5.71 (0.70–46.60）	**5.34 (1.37–20.4)**	1.98 (0.68–5.77)	0.97 (0.10–9.23)	0.56 (0.43–1.22)	1.03 (0.23–4.67)	0.15 (0.02–1.83)
Erlotinib	3.08 (0.46–20.67）	**0.22 (0.06–0.75)**	**0.41 (0.17–0.97)**	0.20 (0.03–1.26)	0.27 (0.05–1.49)	2.60 (0.67–10.10)	0.13 (0.02–1.11)
Gefitinib	2.17 (0.47–10.12)	**0.14 (0.05–0.37)**	**0.25 (0.12–0.50**	0.37 (0.09–1.65)	0.70 (0.20–2.47)	2.63 (1.01–6.85)	0.57 (0.11–2.90)
Icotinib	0.36 (0.03–5.99)	**0.05 (0.01–0.29)**	NA	NA	NA	0.91 (0.15–5.42)	0.06 (0.01–1.94)
Erlotinib+ Bevacizumab	3.14 (0.35–28.20)	0.27 (0.07–1.13)	0.64 (0.23–1.75)	0.26 (0.03–2.13)	NA	1.98 (0.44–8.96)	**0.03 (0.00–0.37)**
Ramucirumab+ Erlotinib	NA	**0.14 (0.03–0.60**	0.79 (0.29–2.17)	NA	0.30 (0.04–2.47)	**5.88 (1.30–26.70)**	**0.05* (0.01–0.60)**
Gefitinib+ PbCT	1.22 (0.19–7.87)	**0.12 (0.04–0.40)**	0.45 (0.17–1.23)	NA	0.44 (0.07–2.57)	**4.21 (1.30–13.50)**	0.59 (0.06–5.87)
Apatinib+Gefitinib	2.07 (0.23–18.3)	NA	0.62 (0.23–1.69)	NA	NA	**3.90 (1.01–15.20)**	NA
PbCT	**0.03 (0.01–0.24)**	**0.02 (0.01–0.09)**	**0.07 (0.02–0.24)**	**0.01 (0.00–0.02)**	**0.04 (0.02–0.08)**	1.39 (0.31–6.32)	0.07 (0.00–1.40)
PfCT	**0.19 (0.03–1.07)**	**0.04 (0.01–0.12)**	**0.10 (0.05–0.23)**	**0.01 (0.00–0.05)**	**0.03 (0.01–0.17)**	0.87 (0.27–2.79)	0.15 (0.02–1.07)
				**versus Afatinib**			
Erlotinib	0.54 (0.11–2.70)	**0.04 (0.01–0.12)**	**0.20 (0.09–0.47)**	0.21 (0.64–1.19)	1.21 (0.89–2.13)	2.50 (0.78–8.13)	0.89 (0.11–7.26)

Gefitinib	0.38 (0.09–1.58)	**0.03 (0.01–0.06)**	**0.12 (0.06–0.28)**	0.38 (0.07–2.08)	1.45 (0.89–2.89)	2.55 (0.79–8.23)	3.91 (0.56–27.2)
Icotinib	0.06 (0.01–0.48)	**0.01 (0.00–0.02)**	NA	NA	NA	0.8 (0.13–5.94)	0.43 (0.04–4.35)
Erlotinib+ Bevacizumab	0.55 (0.08–3.85)	**0.05 (0.01–0.18)**	**0.32 (0.12–0.85)**	0.26 (0.03–2.03)	NA	1.92 (0.50–7.40)	0.22 (0.02–2.42)
Ramucirumab+ Erlotinib	NA	**0.03 (0.01–0.1)**	0.41 (0.15–1.06)	NA	0.67 (0.52–1.87)	**5.71 (1.48–22.10)**	0.36 (0.03–3.96)
Gefitinib+ PbCT	**0.21* (0.05–0.96)**	**0.02 (0.01–0.06)**	**0.23 (0.08–0.67)**	NA	0.89 (0.74–3.51)	**4.07 (1.05–15.70)**	4.03 (0.32–50.20)
Apatinib+Gefitinib	0.36 (0.04–2.95)	NA	**0.31 (0.11–0.91)**	NA	NA	3.8 (0.84–17.20)	NA
PbCT	**0.01 (0.00–0.02)**	**0.01 (0.0–0.02)**	**0.03 (0.02–0.07)**	**0.01 (0.00–0.02)**	**0.04 (0.02–0.08)**	1.35 (0.26–7.10)	0.47 (0.09–2.40)
PfCT	**0.03 (0.01–0.12)**	**0.02 (0.0–0.03)**	**0.05 (0.03–0.11)**	**0.01 (0.00–0.04)**	**0.06 (0.03–0.11)**	0.84 (0.32–2.20)	1.05 (0.21–5.37)
**versus Erlotinib**							
Gefitinib	0.71 (0.23–2.15)	0.63 (0.30–1.30)	0.61 (0.36–1.02)	1.87 (0.63–5.56)	2.61 (0.82–8.35)	1.01 (0.39–2.63)	**4.41 (1.08–18.1)**
Icotinib	0.12 (0.01–1.19)	0.24 (0.05–1.10)	NA	NA	NA	0.35 (0.06–2.08)	0.49 (0.02–11.1)
Erlotinib+ Bevacizumab	1.02 (0.34–3.03)	1.25 (0.62–2.55)	1.58 (0.96–2.60)	1.27 (0.45–3.64)	NA	0.76 (0.39–1.49)	0.35 (0.08–1.03)
Ramucirumab+ Erlotinib	NA	0.66 (0.33–1.34)	1.95 (1.18–3.22)	NA	1.10 (0.31–3.86)	**2.26 (1.15-4.45)**	0.41 (0.13–1.28)
Gefitinib+ PbCT	0.40 (0.09–1.74)	0.55 (0.21–1.43)	1.12 (0.47–2.69)	NA	1.62 (0.29–8.95)	1.61 (0.50–5.20)	4.54 (0.53–39.1)
Apatinib+Gefitinib	0.67 (0.10–4.50)	NA	1.53 (0.64–3.68)	NA	NA	1.51 (0.39–5.83)	NA
PbCT	**0.01 (0.00–0.05)**	**0.11 (0.03–0.33)**	**0.17 (0.06–0.50)**	**0.01 (0.00–0.07)**	**0.07 (0.03–0.33)**	0.54 (0.12–2.43)	0.53 (0.04–7.59)
PfCT	**0.06 (0.02–0.16)**	**0.17 (0.09–0.32)**	**0.25 (0.16–0.4)**	**0.04 (0.02–0.11)**	**0.13 (0.04–0.36)**	0.33 (0.17-0.66)	1.19 (0.31–4.48)
**versus Gefitinib**							
Icotinib	**0.16 (0.04–0.19)**	0.39 (0.10–1.55)	NA	NA	NA	0.34 (0.08–1.56)	0.11 (0.01–2.25)
Erlotinib+ Bevacizumab	1.44 (0.30–6.88)	2.00 (0.73–5.50）	**2.60 (1.27–5.34)**	0.68 (0.15-1.59)	NA	0.75 (0.23–3.42)	**0.06 (0.01–0.35)**
Ramucirumab+ Erlotinib	NA	1.05 (0.38–2.90)	**3.22 (1.57–6.61)**	NA	0.42 (0.08-2.33)	2.23 (0.69–7.21)	**0.09 (0.01–0.57)**
Gefitinib+ PbCT	0.56 (0.20–1.60)	0.88 (0.45–1.70)	1.85 (0.91–3.75)	NA	0.62 (0.18–2.17)	1.59 (0.81–3.13)	1.03 (0.2–5.24)
Apatinib+Gefitinib	0.95 (0.20–4.44)	NA	2.53 (1.25–5.13)	NA	NA	1.49 (0.57–3.88)	NA
PbCT	**0.01 (0.00–0.06)**	**0.17 (0.07–0.44)**	**0.27 (0.09–0.80)**	**0.01 (0.0–0.04)**	**0.04 (0.02–0.09)**	0.53 (0.16–1.71)	0.12 (0.01–1.52)
PfCT	**0.09 (0.04-0.19)**	**0.27 (0.16–0.46)**	**0.42 (0.29–0.61)**	**0.02 (0.01–0.05)**	**0.05 (0.02–0.14)**	**0.33 (0.17–0.65)**	**0.27 (0.09–0.77)**
**versus Icotinib**							
Erlotinib+ Bevacizumab	8.76 (0.67–114.10)	5.14 (0.98–27.10)	NA	NA	NA	2.18 (0.32–14.70)	0.51 (0.02–14.10)
Ramucirumab+ Erlotinib	NA	2.71 (0.51–14.30)	NA	NA	NA	6.49 (0.96–43.4)	0.83 (0.03–23.10)
Gefitinib+ PbCT	3.41 (0.45–26.10)	2.26 (0.61–8.46)	NA	NA	NA	**4.63 (1.2–17.90)**	9.28 (0.30–28.40)
Apatinib+Gefitinib	5.77 (0.41–80.40)	NA	NA	NA	NA	4.30 (0.72–25.60)	NA

PbCT	**0.08 (0.02–0.38)**	0.44 (0.16–1.19)	NA	NA	NA	1.54 (0.59–4.00)	1.09 (0.21–5.50)
PfCT	0.53 (0.06–4.54)	0.71 (0.17–2.85)	NA	NA	NA	0.96 (0.18–6.60)	2.43 (0.14–40.10)
**versus Erlotinib+Bevacizumab**							
Ramucirumab+ Erlotinib	NA	0.53 (0.19–1.43)	1.24 (0.61–2.51)	NA	NA	**2.91 (1.14–7.7)**	1.64 (0.32–8.30)
Gefitinib+ PbCT	0.39 (0.06–2.45)	0.44 (0.13–1.44)	0.71 (0.26–1.95)	NA	NA	2.12 (0.55–8.20)	**18.20 (1.60–20.90)**
Apatinib+Gefitinib	0.66 (0.07–5.90)	NA	0.97 (0.35–2.67)	NA	NA	1.99 (0.44–9.00)	NA
PbCT	**0.01 (0.00–0.07)**	**0.09 (0.02–0.32)**	**0.11 (0.03–0.35)**	**0.01 (0.00–0.07)**	NA	0.71 (0.13–3.70)	2.14 (0.12–38.8)
PfCT	**0.06 (0.01–0.26)**	**0.14 (0.05–0.35)**	**0.16 (0.08–0.32)**	**0.04 (0.01–0.14)**	NA	0.44 (0.17–1.14)	4.77 (0.82–2.77)
**versus Ramucirumab+Erlotinib**							
Gefitinib+ PbCT	NA	0.84 (0.26–2.73)	0.57 (0.21–1.57)	NA	1.47 (0.18–12.20)	0.71 (0.18–2.76)	11.20 (1.70–66.80)
Apatinib+Gefitinib	NA	NA	0.79 (0.29–2.15)	NA	NA	0.67 (0.15–3.02)	NA
PbCT	NA	**0.16 (0.04–0.61)**	**0.09 (0.03–0.28)**	NA	**0.08 (0.03–0.17)**	0.24 (0.05–1.24)	1.31 (0.07–23.7)
PfCT	NA	**0.26 (0.1–0.67)**	**0.13 (0.07–0.25)**	NA	**0.11 (0.02–0.58)**	**0.15 (0.06–0.38)**	2.92 (0.50–16.90)
**versus Gefitinib+PbCT**							
Apatinib+Gefitinib	1.72 (0.22–13.4)	NA	1.37 (0.50–3.71)	NA	NA	0.94 (0.29–3.02)	NA
PbCT	**0.01 (0.00–0.12)**	**0.19 (0.08–0.46)**	**0.15 (0.04–0.53)**	NA	**0.07 (0.03–0.45)**	**0.33 (0.13–0.86)**	0.12 (0.01–2.37)
PfCT	**0.09 (0.02–0.52)**	**0.31 (0.14–0.69)**	**0.23 (0.1–0.51)**	NA	**0.08 (0.01–0.40)**	**0.21 (0.08–0.54)**	0.26 (0.04–1.81)
**versus Apatinib+Gefitinib**							
PbCT	**0.01 (0.0–0.13)**	NA	**0.11 (0.03–0.39)**	NA	NA	0.36 (0.08–1.61)	NA
PfCT	**0.09 (0.02–0.52)**	NA	**0.17 (0.07-0.37)**	NA	NA	**0.22 (0.07–0.29)**	NA
**versus PbCT**							
PfCT	1.52 (0.45–7.40)	1.61 (0.61–4.26)	1.53 (0.56–4.16)	6.30 (0.77–51.70)	1.22 (0.88–4.31)	**0.62 (0.05–0.94)**	2.23 (0.22–22.30)

When not reported, liver dysfunction was represented by alanine transaminase increased as it was reported in most studies. ILD, interstitial lung disease; NA, not available; PbCT, pemetrexed-based chemotherapy; PfCT, pemetrexed-free chemotherapy.

B

**Treatment**	**P-score**						
	**Rash**	**Diarrhea**	**Stomatitis**	**Paronychia**	**Dry skin**	**Liver dysfunction#**	**ILD**
Osimertinib	57%	50%	53%	25%	45%	98%	23%
Dacomitinib	56%	10%	17%	**15%**	**14%**	70%	**12%**
Afatinib	**9%**	**0%**	**2%**	16%	NA	69%	54%
Erlotinib	22%	33%	61%	63%	66%	34%	57%
Gefitinib	33%	54%	79%	43%	22%	34%	22%
Icotinib	75%	79%	NA	NA	NA	73%	72%
Erlotinib+ Bevacizumab	24%	27%	35%	52%	NA	46%	79%
Gefitinib+ PbCT	52%	59%	52%	NA	46%	15%	23%
Ramucirumab+ Erlotinib	NA	53%	25%	NA	58%	**7%**	77%

Apatinib+ Gefitinib	36%	NA	37%	NA	NA	19%	NA
PbCT	100%	98%	98%	99%	95%	57%	66%
PfCT	87%	89%	92%	86%	100%	77%	54%

When not reported, liver dysfunction was represented by alanine transaminase increased as it was reported in most studies. ILD, interstitial lung disease; NA, not available; PbCT, pemetrexed-based chemotherapy; PfCT, pemetrexed-free chemotherapy.

Table S4. Bayesian ranking results of the network meta-analysis in the first sensitivity analysis.

Treatment	Rank of Possibility (%)											
	1	2	3	4	5	6	7	8	9	10	11	12
**Overall survival**												
Osimertinib	38.1	26.7	15.4	8.6	4.3	1.6	1.6	1.4	0.6	0.5	NA	NA
Dacomitinib	2.7	10.6	22.5	27.2	15.3	9.7	5.6	4.2	1.5	0.8	NA	NA
Afatinib	**39.2**	24.6	14.6	13.5	4.2	1.0	0.2	0	0	0	NA	NA
Erlotinib	0	0	0	0	0.3	12.5	10.7	19.4	42.6	25.4	NA	NA
Gefitinib	0	0.2	2.7	14.9	32.9	23.6	15.6	8.0	1.7	0.4	NA	NA
Icotinib	2.2	5.4	10.1	14.3	11.8	10.7	16.2	11.3	10.8	7.2	NA	NA
Erlotinib+ Bevacizumab	0	0.2	0.7	1.6	2.5	4.0	9.9	13.8	22.6	**44.5**	NA	NA
Gefitinib+ PbCT	17.1	32.1	31.0	8.6	4.6	2.9	2.0	1.8	0.9	0.8	NA	NA
Ramucirumab+ Erlotinib	NA	NA	NA	NA	NA	NA	NA	NA	NA	NA	NA	NA
Apatinib+ Gefitinib	NA	NA	NA	NA	NA	NA	NA	NA	NA	NA	NA	NA
PbCT	0	0.2	1.5	4.6	8.2	9.2	13.7	24.5	17.8	20.2	NA	NA
PfCT	0	0.2	1.5	6.5	15.6	35.0	24.3	15.5	1.4	0	NA	NA
**Progression-free survival**												
Osimertinib	6.9	23.1	38.4	20.3	8.2	2.6	0.5	0	0	0	0	0
Dacomitinib	6.1	13.2	20.4	30.3	18.0	8.3	3.0	0.7	0	0	0	0
Afatinib	0.6	1.5	2.9	5.5	10.0	15.9	23.1	32.0	8.4	0	0	0
Erlotinib	0	0	0.5	5.8	15.5	24.4	29.8	22.2	1.6	0	0	0
Gefitinib	0	0	0	0	0	0	0.5	11.5	83.5	4.4	0	0
Icotinib	0	0	0	0	0	0	0.2	0.8	3.5	85.6	7.5	2.2
Erlotinib+ Bevacizumab	**67.3**	17.6	7.3	4.1	2.1	1.0	0.4	0	0	0	0	0
Gefitinib+ PbCT	1.8	4.1	7.3	13.5	23.1	22.9	0.4	0	0	0	0	0
Ramucirumab+ Erlotinib	16.2	38.3	19.1	12.7	7.9	4.2	1.5	0.1	0	0	0	0
Apatinib+ Gefitinib	0.9	2.2	4.0	7.9	15.1	20.5	23.3	23.4	2.6	0.2	0	0
PbCT	0	0	0	0	0	0.1	0.5	1.4	3.5	3.5	68.1	28.3
PfCT	0	0	0	0	0	0	0	0	0	6.2	24.3	**69.4**
**Adverse events of grade ≥3**												
Osimertinib	3.1	49.8	30.9	10.9	4.0	1.0	0.2	0	0	0	0	0
Dacomitinib	0.7	10.6	10.1	15.8	33.9	15.6	8.4	4.0	0.8	0	0	0
Afatinib	0	0.3	0.7	1.8	8.4	36.1	31.1	17.1	4.4	0	0	0
Erlotinib	1.6	24.0	33.1	19.8	14.9	4.5	1.6	0.4	0	0	0	0
Gefitinib	0.1	5.0	18.6	44.3	25.3	5.4	1.2	0.1	0	0	0	0
Icotinib	**94.4**	2.2	1.5	0.9	0.9	0.1	0	0	0	0	0	0
Erlotinib+ Bevacizumab	0	0	0	0	0	0	0	0.4	1.8	8.4	54.9	34.4
Gefitinib+ PbCT	0	4.7	2.4	2.7	4.3	9.4	11.4	18.8	25.7	12.6	5.2	2.7
Ramucirumab+ Erlotinib	0	0	0	0.4	2.5	14.1	24.1	27.0	30.9	0.9	0	0
Apatinib+ Gefitinib	0	0	0	0	0	0	0	0	0.3	2.6	34.4	**62.7**
PbCT	0	3.3	2.7	3.3	5.8	13.8	21.6	29.2	16.9	3.6	0.9	0.1
PfCT	0	0	0	0	0	0	0	3.8	19.5	72.1	4.6	0

*The numbers with biggest probability of ranking first and last are in bold and underscored. NA, not available; PbCT, pemetrexed-based chemotherapy; PfCT, pemetrexed-free chemotherapy.

Table S5. Bayesian node-splitting analysis of inconsistency in the first sensitivity analysis.

Nodes	Direct Effect	Indirect Effect	Overall	P
**Progression-free survival**				
Afatinib, PbCT	0.27 (0.17–0.58)	0.15 (0.09–0.28)	0.60 (0.34–1.07)	0.381
Afatinib, PfCT	0.20 (0.09–0.42)	0.38 (0.18–0.55)	0.41 (0.23–0.75)	0.394
Erlotinib, Osimertinib	1.69 (0.96–2.34)	0.94 (0.88–1.66)	1.96 (1.20–3.13)	0.219
Erlotinib, PfCT	0.16 (0.08–0.28)	0.31 (0.16–0.37)	0.41 (0.26–0.64)	0.225
Gefitinib, Gefitinib+PbCT	1.53 (1.08–2.16)	2.93 (1.70–3.61)	2.64 (1.34–5.86)	0.404
Gefitinib, Osimertinib	1.91 (1.20–2.66)	3.44 (1.82–4.31)	1.86 (1.23–2.78)	0.211
Gefitinib, PfCT	0.33 (0.21–0.50)	0.18 (0.12–0.24)	0.39 (0.28–0.54)	0.097
Gefitinib+PbCT, PbCT	0.15 (0.09–0.26)	0.27 (0.17–0.52)	0.21 (0.10–0.46)	0.374
**Adverse events of grade ≥3**				
Afatinib, PbCT	1.03 (0.39–2.74)	0.24 (0.18–0.88)	0.83 (0.34–2.08)	0.353
Afatinib, PfCT	0.37 (0.23–0.58)	1.33 (0.36–1.41)	0.39 (0.25–0.61)	0.363
Erlotinib, Osimertinib	1.38 (0.89–1.17)	0.79 (0.55–1.23)	1.01 (0.74–1.63)	0.198
Erlotinib, PfCT	0.19 (0.06–0.52)	0.16 (0.08–0.33)	0.22 (0.14–0.34)	0.201
Gefitinib, Gefitinib+PbCT	0.16 (0.07–0.48)	0.78 (0.22–1.57)	0.42 (0.11–1.61)	0.370
Gefitinib, Osimertinib	0.89 (0.64–3.21)	2.50 (1.21–3.11)	1.23 (0.87–1.75)	0.212
Gefitinib, PfCT	0.26 (0.18–0.42)	0.09 (0.08–0.27)	0.24 (0.19–0.30)	0.097
Gefitinib+PbCT, PbCT	0.80 (0.20–3.22)	4.22 (1.21–6.45)	1.23 (0.37–4.00)	0.374

* The Bayesian node-splitting analysis was applied to evaluate inconsistency for closed loops in the network. No closed loop is found in the network of overall survival. PbCT, pemetrexed-based chemotherapy; PfCT, pemetrexed-free chemotherapy.

A

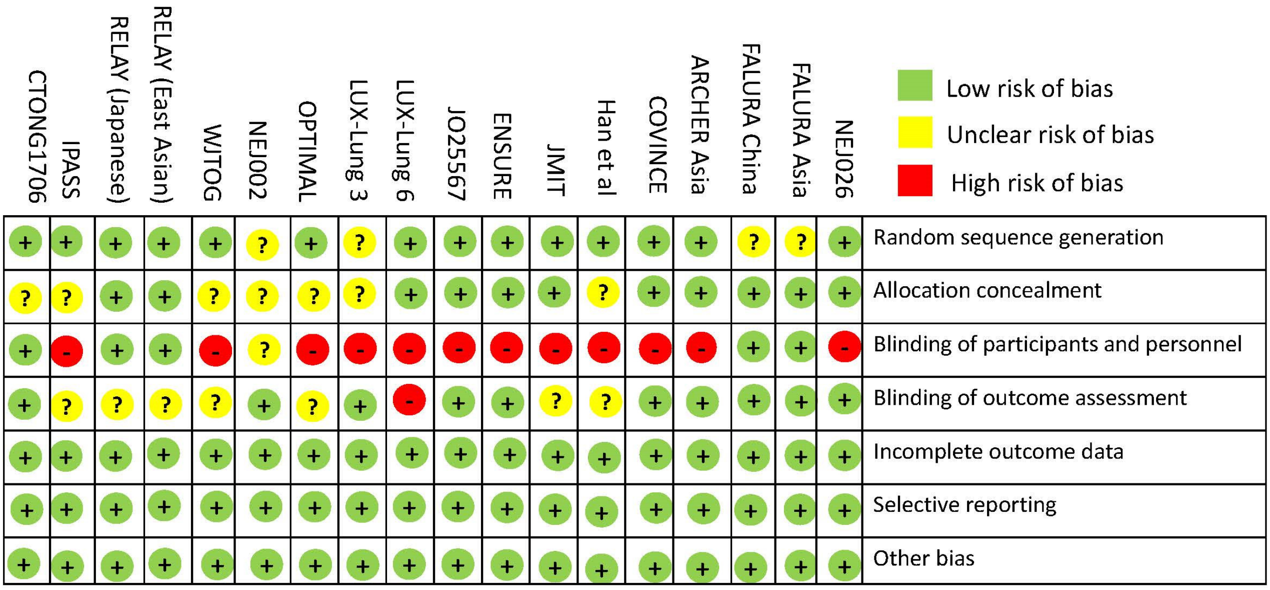

B

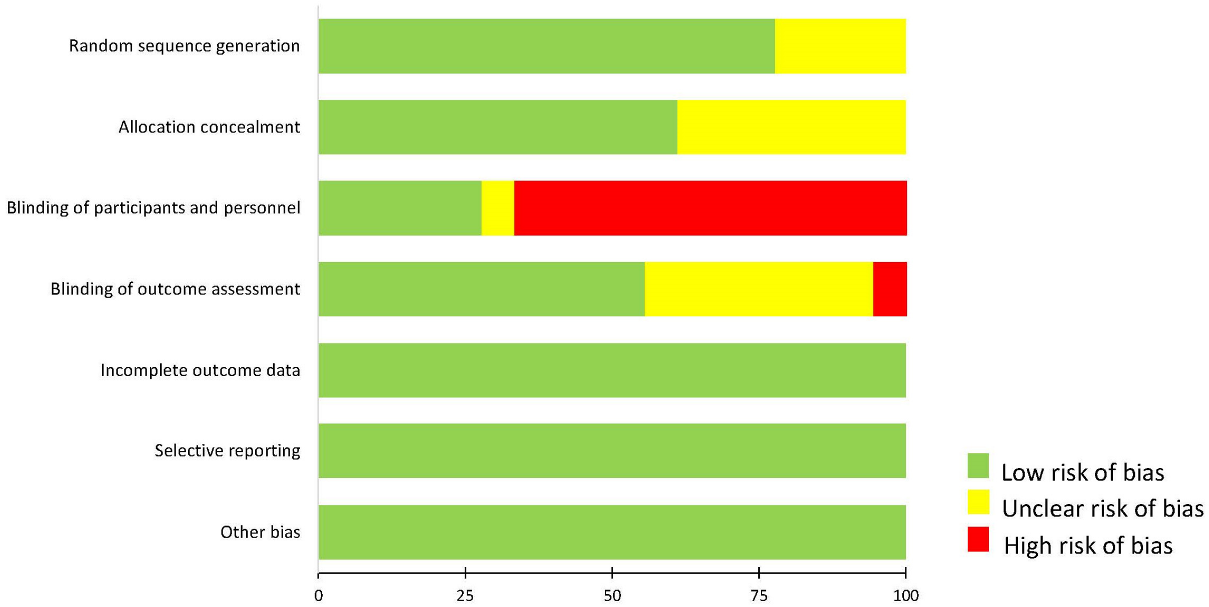

Figure S1. (**A**) Summary of the risk of bias assessment in 7 domains in the 18 studies included in the network meta-analysis. (**B**) Graphical representation of the overall risk of bias in the 7 domains.

A

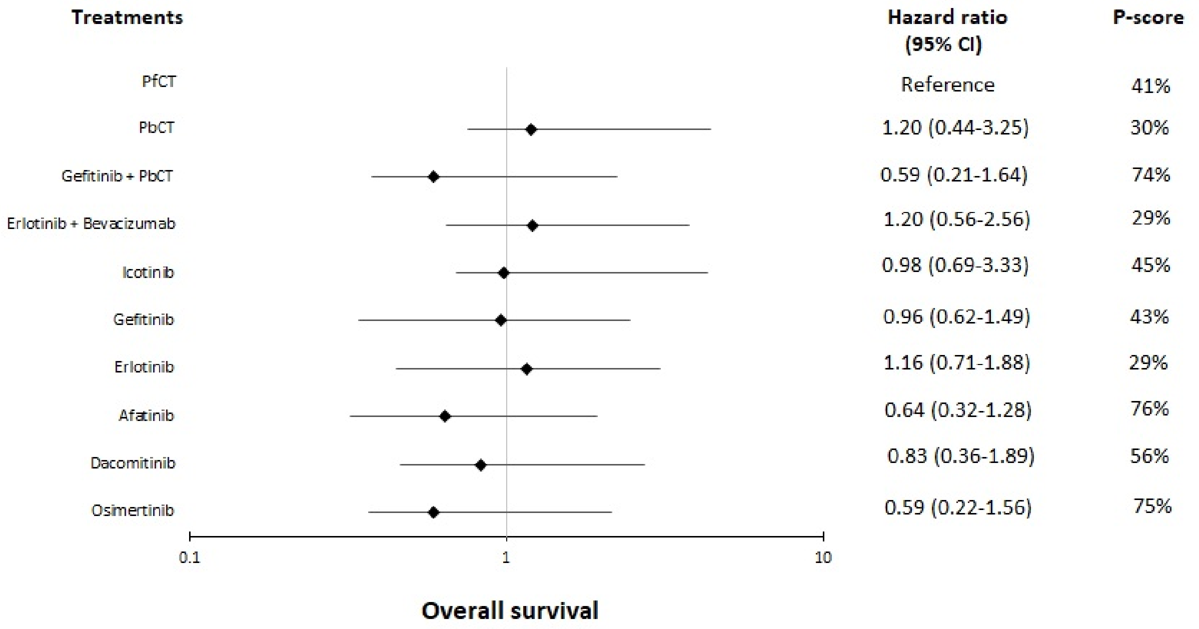

B

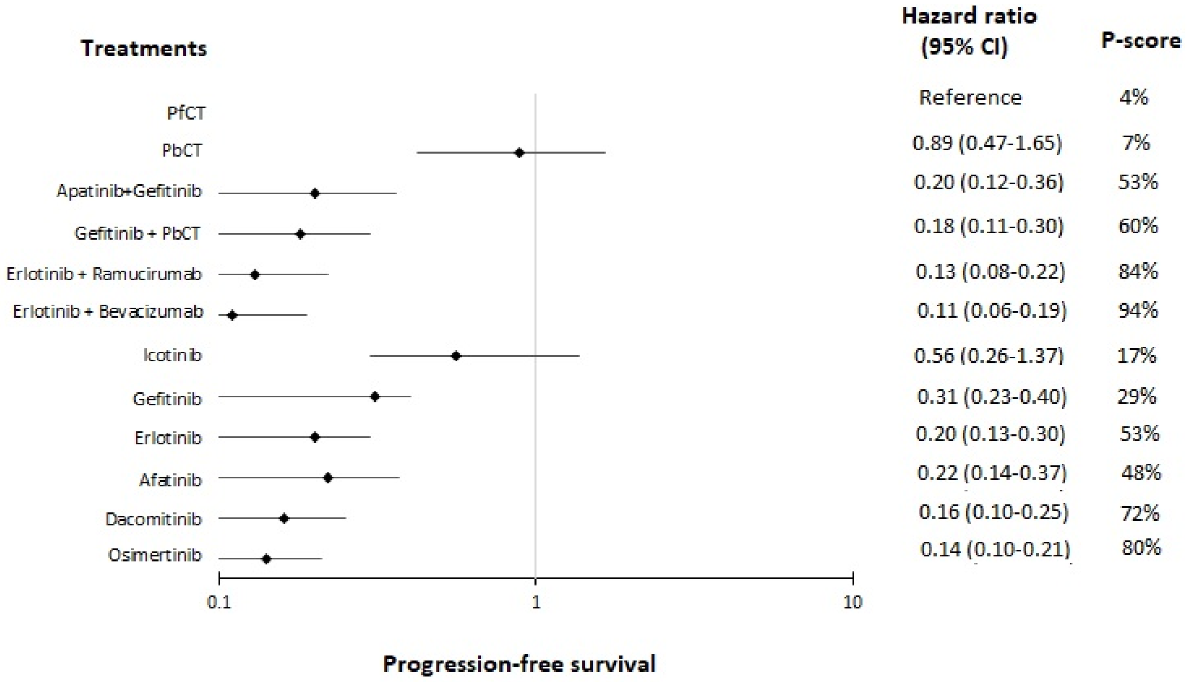

Figure S2. Forest plot for (**A**) overall survival and (**B**) progression-free survival showing results comparing treatment regimens against pemetrexed-free chemotherapy from the network meta-analysis. Hazard ratios more than 1 favor pemetrexed-free chemotherapy. EGFR, epidermal growth factor receptor; TKIs, tyrosine kinase inhibitors; PbCT, pemetrexed-based chemotherapy; PfCT, pemetrexed-free chemotherapy; 95% CI, 95% confidence interval.

A

Progression-free survival

Overall survival (rows)

**Third-generation TKIs (71%; 84%)**	0.61 (0.32–1.17)	**0.54 (0.34–0.85)**	0.89 (0.44–1.81)	0.88 (0.49–1.57)	**0.16 (0.09–0.29)**
1.12 (0.45–2.81)	**Second-generation TKIs (73%; 41%)**	0.88 (0.56–1.38)	1.45 (0.73–2.87)	1.43 (0.80–2.55)	**0.26 (0.18–0.40)**
1.64 (0.72–3.74)	1.46 (0.98–2.19)	**First-generation TKIs (29%; 26%)**	1.66 (0.96–2.85)	**1.62 (1.13–2.34)**	**0.30 (0.22–0.42)**
1.00 (0.30–3.33)	0.89 (0.34–2.34)	0.61 (0.25–1.46)	**First-generation TKIs+ chemotherapy (73%; 69%)**	0.98 (0.51–1.89)	**0.18 (0.10–0.33)**
1.69 (0.63–14.56)	1.51 (0.77–2.98)	1.03 (0.60–1.78)	1.69 (0.60–4.75)	**First-generation TKIs+ antiangiogenic agents (29%; 78%)**	**0.19 (0.11–0.30)**
1.56 (0.71–4.01)	1.51 (0.98–2.20)	1.03 (0.80–1.33)	1.69 (0.69–4.21)	1.00 (0.55–1.82)	**Chemotherapies (23%; 0%)**

B

Progression-free survival

Overall survival (rows)

**Irreversible TKIs (94%; 87%)**	0.98 (0.68–1.33)	**0.58 (0.45–0.74)**	**0.20 (0.15–0.26)**
1.34 (0.76–2.36)	**Combination treatments with reversible TKIs (52%; 79%)**	**0.61 (0.48–0.77)**	**0.21 (0.15–0.28)**
**1.50 (1.02–2.12)**	1.12 (0.71–1.76)	**Reversible TKIs (30%; 33%)**	**0.34 (0.27–0.42)**
1.53 (0.98–2.15)	1.15 (0.69–1.91)	0.88 (0.75–1.05)	**Chemotherapies (24%; 0%)**

Figure S3. Pooled estimates of the exploratory analyses for overall survival and progression-free survival. (**A**) Pooled hazard ratios (95% confidence intervals) for overall survival (upper triangle) and progression-free survival (lower triangle) stratified by the generation of treatment regimens. (**B**) Pooled hazard ratios (95% confidence intervals) for overall survival (upper triangle) and progression-free survival (lower triangle) stratified by the reversibility of the treatment regimens. P-scores for overall survival (left) and progression-free survival (right) are indicated under each treatment. (Data in each cell are hazard ratios (95% confidence intervals) for the comparison of row-defining treatment versus column-defining treatment. Hazard ratios less than 1 favor row-defining treatment. Significant results are in bold.

A

Adverse events of grade 3 or above

**Third-generation TKIs (89%)**					
**2.72 (1.01–7.41)**	**Second-generation TKIs (50%)**				
0.99 (0.61–1.61)	**0.36 (0.15–0.88)**	**First-generation TKIs (90%)**			
**4.68 (1.13–19.38)**	1.72 (0.38–7.79)	**4.73 (1.24–17.98)**	**First-generation TKIs+ chemotherapy (25%)**		
**3.94 (1.94–7.99)**	1.45 (0.52–4.02)	**3.98 (2.38–6.66)**	0.84 (0.20–3.53)	**First-generation TKIs+ antiangiogenic agents (32%)**	
**5.11 (2.62–9.97)**	1.88 (0.89–3.97)	**5.16 (3.26–8.18)**	1.09 (0.29–4.05)	1.30 (0.65–2.59)	**Chemotherapies (15%)**

B

Adverse events of grade 3 or above

**Irreversible TKIs (71%)**			
**3.13 (1.63–6.00)**	**Combination treatments with reversible TKIs (18%)**		
0.78 (0.51–1.21)	**0.25 (0.15–0.41)**	**Reversible TKIs (96%)**	
**3.26 (1.96–5.43)**	1.04 (0.56–1.93)	**4.18 (2.77–6.32)**	**Chemotherapies (15%)**

Figure S4. Pooled estimates of the exploratory analyses for adverse events of grade ≥ 3. (**A**) Pooled odds ratios (95% confidence intervals) stratified by the generation of treatment regimens. (**B**) Pooled odds ratios (95% confidence intervals) stratified by the reversibility of the treatment regimens. P-scores are indicated under each treatment. (Data in each cell are odds ratios (95% confidence intervals) for the comparison of row-defining treatment versus column-defining treatment. Hazard ratios less than 1 favor row-defining treatment. Significant results are in bold.

Adverse events	Rash	Diarrhoea	Stomatitis	Paronychia	Dry skin	Pruritus	Anorexia	Fatigue	Constipation	Nausea	Vomitting	Leucopenia	Neutropenia	Anaemia	Liver dysfunction*	ILD
No.of studies	15	18	15	11	9	10	13	13	12	14	12	10	13	15	15	14
Sample size	4300	4987	4270	3583	1703	3487	3970	2874	3653	4030	3647	3110	3566	4103	3387	3204
Osimertinib	50.6	51.5	29.2	30.9	27.9	14.8	18	4.9	16.7	10.7	14.1	10.3	9.9	21.5	8.5	4.7
Dacomitinib	21.2	90.6	51.2	64.7	24.7	18.2	33.5	10.6	14.7	18.2	8.2	NA	NA	10.6	24.7	2.9
Afatinib	84.3	90.4	59	43.7	46.3	12.6	15.7	13	2.4	10.6	11.6	3.8	2	4.8	20.1	1
Erlotinib	81.8	58.7	37.8	22.9	29.3	21.9	15.3	3.4	6.3	7.8	5.6	6.4	3.9	4.6	31.2	3.1
Gefitinib	63.8	49.8	18.2	17.6	30.4	16	21.4	13.1	12.2	16.4	11.1	3.8	6.3	9.7	49	2.1
Icotinib	15.5	9.5	NA	NA	NA	1.4	4.1	NA	NA	5.4	2.7	9.5	6.1	5.4	9.5	0
Erlotinib+Bev	92	61	37.4	39.6	71	45.3	20.9	5.9	22.7	16	18.7	NA	1.1	5.4	19.3	1.1
Erlotinib+Ram	NA	77.6	54.7	NA	59.3	NA	NA	NA	NA	NA	NA	NA	NA	NA	58.8	1.6
Gefitinib+PbCT	61	45.2	35.7	NA	25.4	NA	41	26.5	47.5	33.1	12.7	20	42.5	24.1	41.6	1.6
Gefitinib+Apatinib	55.4	73.2	NA	NA	NA	9.6	42.7	28.7	17.2	28.1	28.7	NA	NA	NA	55.4	NA
PbCT	3.9	8.7	25	0	3.6	1.2	34.5	36.2	50.7	52.4	33.3	42.7	44.7	22.3	16.9	0
PfCT	18.4	18.1	7.2	0	2.6	8.1	42.5	37.1	25	54	39.8	47.3	28.9	31.9	23.5	0.2

Figure S5. Toxicity profile in relation to the incidence (%) of each specific adverse event (any grade) based on the overall population of each treatment included in the network meta-analysis. *When not reported, liver dysfunction was represented by alanine transaminase increased as it was reported in most studies. Notable incidences of hypertension (58.3%), hemorrhagic events (45.5%) and proteinuria (40.1%) were also associated with erlotinib plus bevacizumab group based on the report of the JO25567 and NEJ026 studies. Notable incidence of hypertension (46.5%) was associated with gefitinib plus apatinib based on CTONG1706. Bev, bevacizumab; ILD, interstitial lung disease; NA, not available; PbCT, pemetrexed-based chemotherapy; PfCT, pemetrexed-free chemotherapy; Ram, ramucirumab.

A

Progression-free survival

Overall survival

**Osimertinib**	0.90 (0.54–1.51)	0.62 (0.32–1.17)	0.71 (0.48–1.05)	**0.45 (0.33–0.66)**	**0.24 (0.10–0.59)**	1.32 (0.74–2.36)	1.08 (0.63–1.85)	0.77 (0.45–1.31)	0.69 (0.38–1.26)	**0.16 (0.08–0.32)**	**0.14 (0.10–0.21)**
1.40 (0.45–4.30)	**Dacomitinib**	0.70 (0.36–1.37)	0.80 (0.44–1.39)	**0.51 (0.35–0.76)**	0.26 (0.10–0.67)	1.47 (0.72–2.99)	1.21 (0.60–2.36)	0.85 (0.49–1.50)	0.77 (0.41–1.43)	**0.18 (0.10–0.38)**	**0.16 (0.10–0.25)**
1.09 (0.31–3.68)	0.73 (0.24–2.29)	**Afatinib**	1.12 (0.60–2.10)	0.73 (0.42–1.06)	**0.40 (0.20–0.78)**	2.09 (0.58–4.47)	1.71 (0.81–3.53)	1.21 (0.65–2.26)	1.09 (0.52–2.28)	**0.25 (0.15–0.41)**	**0.22 (0.14–0.37)**
1.98 (0.66–5.94)	1.42 (0.54–3.67)	1.82 (0.77–4.28)	**Erlotinib**	**0.65 (0.44–0.99)**	0.34 (0.13–0.84)	**1.86 (1.21–2.86)**	**1.51 (1.10–2.21)**	1.08 (0.61–1.92)	0.97 (0.51–1.85)	**0.21 (0.09–0.46)**	**0.20 (0.13–0.30)**
1.64 (0.68–3.94)	1.17 (0.58–2.34)	1.50 (0.66–3.43)	0.83 (0.43–1.59)	**Gefitinib**	**0.65 (0.43–0.99)**	**2.86 (1.57–5.19)**	**2.34 (1.34–4.08)**	**1.60 (1.11–2.50)**	1.50 (0.90–2.44)	**0.34 (0.18–0.66)**	**0.31 (0.23–0.41)**
1.70 (0.31–8.02)	1.20 (0.21–5.31)	1.55 (0.51–4.28)	0.84 (0.23–3.17)	1.02 (0.28–3.76)	**Icotinib**	**5.55 (2.02–15.23)**	**4.54 (1.69–12.16)**	**3.21 (1.36–7.67)**	**2.90 (1.09–7.44)**	0.67 (0.38–1.17)	0.56 (0.26–1.37)
2.03 (0.61–7.09)	1.46 (0.48–4.47)	1.88 (0.67–5.29)	1.03 (0.58–1.85)	1.25 (0.52–3.01)	1.22 (0.29–5.19)	**Erlotinib+ Bevacizumab**	0.82 (0.46–1.44)	0.58 (0.28–1.19)	0.52 (0.24–1.13)	**0.12 (0.08–0.28)**	**0.11 (0.06–0.19)**
NA	NA	NA	NA	NA	NA	NA	**Ramucirumab+ Erlotinib**	0.71 (0.36–1.41)	0.64 (0.32–1.34)	**0.15 (0.07–0.33)**	**0.13 (0.09–0.22)**
1.00 (0.30–5.37)	0.71 (0.22–2.26)	0.92 (0.27–3.16)	0.50 (0.16–1.57)	0.62 (0.21–1.55)	0.60 (0.12–2.95)	0.49 (0.14–1.75)	NA	**Gefitinib+ PbCT**	0.90 (0.47–1.70)	**0.21 (0.11–0.40)**	**0.18 (0.11–0.31)**
NA	NA	NA	NA	NA	NA	NA	NA	NA	**Apatinib+ Gefitinib**	**0.23 (0.10–0.52)**	**0.20 (0.12–0.36)**
2.11 (0.48–8.08)	1.44 (0.40–5.21)	1.88 (0.80–3.74)	1.02 (0.34–3.09)	1.23 (0.42–3.65)	1.20 (0.59–2.48)	0.99 (0.28–3.45)	NA	2.02 (0.49–8.40)	NA	**PbCT**	0.89 (0.47–1.65)
1.70 (0.64–4.54)	1.21 (0.53–2.75)	1.56 (0.78–3.14)	0.86 (0.53–1.40)	1.04 (0.67–1.61)	1.02 (0.30–3.47)	0.84 (0.40–1.79)	NA	1.72 (0.58–4.70)	NA	0.84 (0.31–2.28)	**PfCT**

B

**Osimertinib (80%)**											
1.23 (0.47–3.21)	**Dacomitinib (71%)**										
2.09 (0.80–2.49)	1.71 (0.53–5.49)	**Afatinib (49%)**									
1.01 (0.54–1.89)	0.82 (0.53–2.34)	0.48 (0.18–1.28)	**Erlotinib (79%)**								
1.10 (0.64–1.88)	0.89 (0.40–1.98)	0.52 (0.22–1.23)	1.08 (0.55–2.14)	**Gefitinib (77%)**							
0.36 (0.07–1.85)	0.29 (0.05–1.70)	**0.17 (0.04–0.7)**	0.36 (0.07–1.86)	0.33 (0.07–1.58)	**Icotinib (94%)**						
**8.33 (3.28–21.19)**	**6.79 (1.94–23.77)**	**3.98 (1.20–13.18)**	**8.25 (4.12–16.49)**	**7.61 (2.88–20.05)**	**23.01 (3.86–137.1)**	**Erlotinib+ Bevacizumab (10%)**					
**2.36 (1.02–5.47)**	1.92 (0.58–6.29)	1.13 (0.36–3.47)	**2.33 (1.33–4.1)**	2.15 (0.89–5.19)	**6.51 (1.14–37.0)**	**0.28 (0.12–0.69)**	**Ramucirumab+ Erlotinib (47%)**				
3.02 (0.62–14.49)	2.46 (0.45–13.49)	1.44 (0.33–6.3)	2.99 (0.6–14.94)	**2.76 (1.00–12.42)**	**8.33 (1.7–40.97)**	0.36 (0.06–2.09)	1.28 (0.23–7.05)	**Gefitinib+ PbCT (25%)**			
**9.42 (3.42–25.87)**	**7.79 (2.39–25.37)**	**4.13 (1.24–13.83)**	**9.30 (3.11–27.83)**	**8.73 (3.76–20.26)**	**20.85 (3.72–116.9)**	1.13 (0.30–4.17)	**3.99 (1.15–13.84)**	1.99 (0.54–7.35)	**Apatinib+ Gefitinib (7%)**		
2.55 (0.66–9.87)	2.07 (0.46–9.31)	1.22 (0.42–3.51)	2.52 (0.64–9.94)	2.32 (0.65–8.31)	**7.03 (2.84–17.39)**	0.31 (0.07–1.42)	1.08 (0.25–4.76)	0.84 (0.23–3.12)	2.97 (0.70–12.62)	**PbCT (38%)**	
**5.10 (2.69–9.66)**	**4.15 (1.65–10.45)**	**2.43 (1.17–5.08)**	**5.04 (2.63–99.67)**	**4.65 (2.91–7.43)**	**14.07 (3.07–64.56)**	0.61 (0.24–1.58)	2.16 (0.91–5.11)	1.69 (0.38–7.58)	1.82 (0.69–4.81)	2.00 (0.59–6.81)	**PfCT (22%)**

Figure S6. Pooled estimates of the network meta-analysis in the first sensitivity analysis by Bayesian approach. (**A**) Pooled hazard ratios (95% credible intervals) for overall survival (upper triangle) and progression-free survival (lower triangle). (**B**) Pooled odds ratios (95% credible intervals) for adverse events of grade ≥3. Data in each cell are hazard or odds ratios (95% credible intervals) for the comparison of row-defining treatment versus column-defining treatment. Hazard ratios or odds ratio less than 1 favor row-defining treatment. Significant results are in bold. NA, not available; PbCT, pemetrexed-based chemotherapy; PfCT, pemetrexed-free chemotherapy.

A

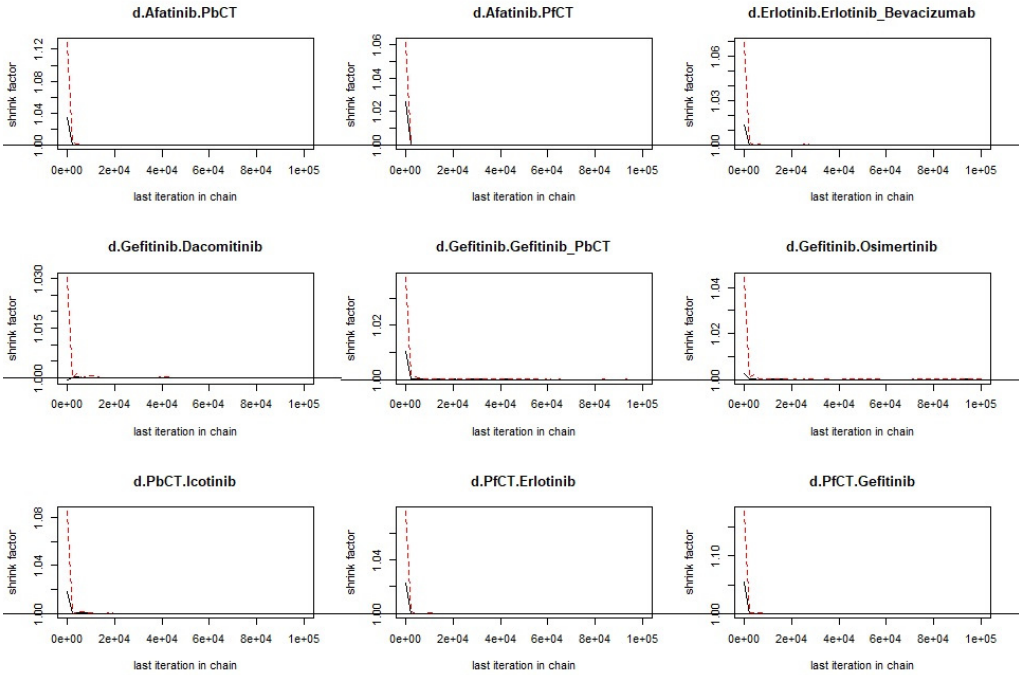
d.Afatinib.PbCT
d.Afatinib.PfCT
d.Erlotinib.Erlotinib_Bevacizumab
d.Gefitinib.Dacomitinib
d.Gefitinib.Gefitinib_PbCT
d.Gefitinib.Osimertinib
d.PbCT.Icotinib
d.PfCT.Erlotinib
d.PfCT.Gefitinib
shrink factor
last iteration in chain
0e+00 2e+04 4e+04 6e+04 8e+04 1e+05

B

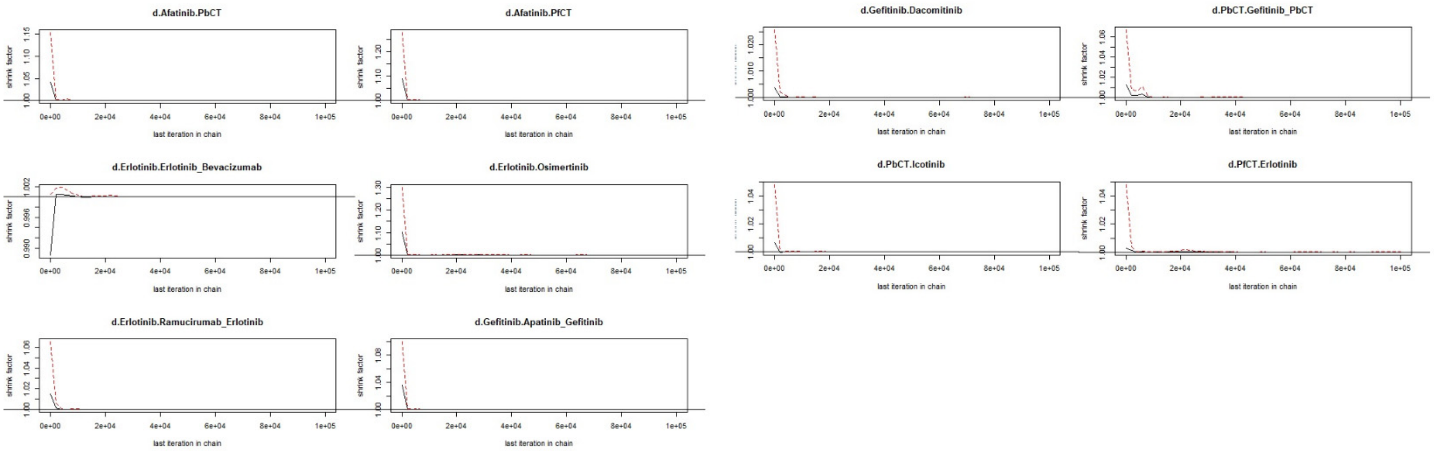
d.Afatinib.PbCT
d.Afatinib.PfCT
d.Gefitinib.Dacomitinib
d.PbCT.Gefitinib_PbCT
d.Erlotinib.Erlotinib_Bevacizumab
d.Erlotinib.Osimertinib
d.PbCT.Icotinib
d.PfCT.Erlotinib
d.Erlotinib.Ramucirumab_Erlotinib
d.Gefitinib.Apatinib_Gefitinib
shrink factor
last iteration in chain

C

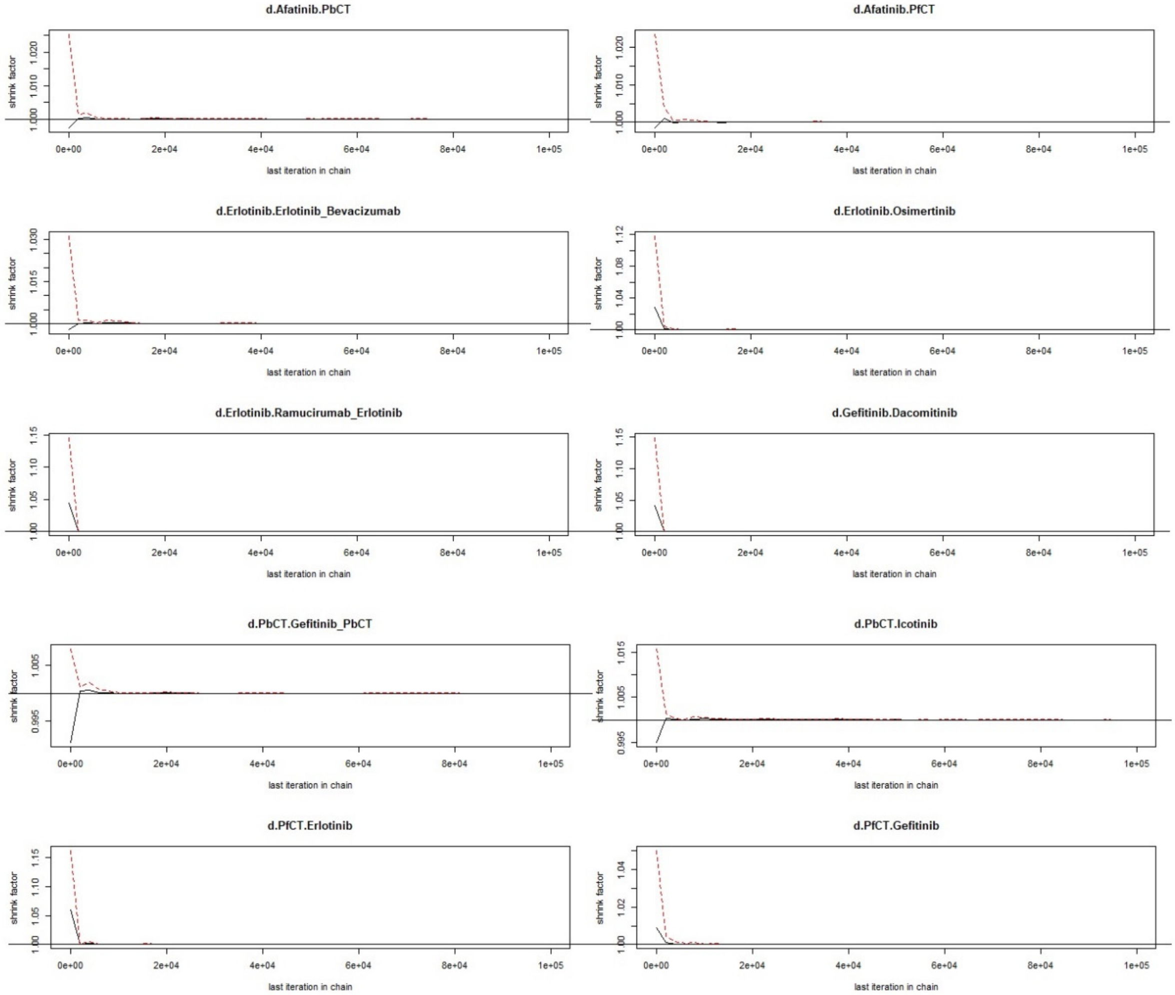

Figure S7. Convergence of the three chains established by inspection of the history feature and the Brooks-Gelman-Rubin diagnostic for (**A**) overall survival, (**B**) progression-free survival, and (**C**) adverse events of grade ≥ 3.

A

Progression-free survival

Overall survival

**Osimertinib (76%; 80%)**	0.90 (0.54–1.51)	0.63 (0.34–1.18)	0.71 (0.48–1.05)	**0.46 (0.33–0.65)**	**0.24 (0.10–0.59)**	1.32 (0.74–2.36)	1.08 (0.63–1.85)	0.77 (0.45–1.31)	0.69 (0.38–1.26)	**0.16 (0.08–0.32)**	**0.14 (0.10–0.21)**
1.40 (0.46–4.30)	**Dacomitinib (56%; 72%)**	0.70 (0.36–2.10)	0.79 (0.45–1.39)	**0.51 (0.35–0.76)**	**0.26 (0.10–0.67)**	1.47 (0.72–2.99)	1.20 (0.61–2.36)	0.85 (0.49–1.50)	0.77 (0.41–1.43)	**0.18 (0.08–0.38)**	**0.16 (0.10–0.25)**
1.09 (0.33–3.63)	0.78 (0.26–2.28)	**Afatinib (77%; 48%)**	1.12 (0.60–2.12)	0.73 (0.42–1.06)	**0.38 (0.18–0.79)**	2.09 (0.58–4.47)	1.71 (0.82–3.54)	1.21 (0.65–2.26)	1.09 (0.52–2.28)	**0.25 (0.15–0.41)**	**0.22 (0.14–0.37)**
1.98 (0.66–5.94)	1.41 (0.54–3.67)	1.82 (0.77–4.28)	**Erlotinib (29%; 53%)**	**0.65 (0.43–0.99)**	**0.34 (0.13–0.84)**	**1.86 (1.21–2.86)**	**1.52 (1.05–2.20)**	1.08 (0.61–1.92)	0.97 (0.51–1.85)	**0.22 (0.11–0.46)**	**0.20 (0.13–0.30)**
0.98 (0.56–-1.70)	1.17 (0.58–2.34)	1.50 (0.66–3.43)	0.83 (0.43–1.59)	**Gefitinib (43%; 29%)**	0.51 (0.22–1.21)	**2.86 (1.57–5.19)**	**2.34 (1.34–4.08)**	**1.66 (1.10–2.50)**	1.49 (0.91–2.44)	**0.34 (0.18–0.66)**	**0.31 (0.23–0.40)**
1.68 (0.35–8.06)	1.19 (0.27–5.22)	1.54 (0.56–4.22)	0.84 (0.23–3.17)	1.02 (0.28–3.76)	**Icotinib (45%; 17%)**	**5.55 (2.02–15.23)**	**4.54 (1.69–12.16)**	**3.22 (1.36–7.66)**	**2.90 (1.09–7.74)**	0.67 (0.38–1.17)	0.56 (0.26–1.37)
2.05 (0.59–-7.09)	1.46 (0.48–4.47)	1.88 (0.67–5.29)	1.03 (0.58–1.85)	1.25 (0.52–3.01)	0.74 (0.29–1.90)	**Erlotinib+ Bevacizumab (29%; 94%)**	0.82 (0.46–1.44)	0.58 (0.28–1.19)	0.52 (0.24–1.13)	**0.12 (0.05–0.28)**	**0.11 (0.06–0.19)**
NA	NA	NA	NA	NA	NA	NA	**Ramucirumab+ Erlotinib (NA; 84%)**	0.71 (0.36–1.41)	0.64 (0.30–1.34)	**0.15 (0.07–0.33)**	**0.13 (0.08–0.22)**
1.00 (0.28–3.58)	0.71 (0.22–2.26)	0.92 (0.27–3.16)	0.50 (0.16–1.57)	0.61 (0.24–1.54)	1.22 (0.29–5.19)	0.49 (0.14–1.75)	NA	**Gefitinib+ PbCT (75%; 60%)**	0.90 (0.47–1.70)	**0.21 (0.11–0.40)**	**0.18 (0.11–0.30)**
NA	NA	NA	NA	NA	NA	NA	NA	NA	**Apatinib+ Gefitinib (NA; 53%)**	**0.23 (0.10–0.52)**	**0.20 (0.12–0.36)**
2.02 (0.50–8.15)	1.44 (0.40–5.21)	1.85 (0.91–3.75)	1.02 (0.34–3.09)	1.23 (0.42–3.65)	1.20 (0.59–2.48)	0.99 (0.28–3.45)	NA	2.20 (0.49–8.40)	NA	**PbCT (30%; 7%)**	0.89 (0.47–1.65)
1.70 (0.64–4.54)	1.21 (0.53–2.75)	1.56 (0.78–3.14)	0.86 (0.53–1.40)	1.04 (0.67–1.61)	1.02 (0.30–3.47)	0.83 (0.39–1.79)	NA	1.70 (0.61–4.47)	NA	0.84 (0.31–3.75)	**PfCT (41%; 4%)**

B

Adverse events of grade 3 or above

**Osimertinib (74%)**											
1.22 (0.44–3.40)	**Dacomitinib (63%)**										
1.83 (0.63–5.32)	1.50 (0.41–5.42)	**Afatinib (46%)**									
0.98 (0.50–1.92)	0.81 (0.26–2.46)	0.54 (0.19–1.56)	**Erlotinib (74%)**								
1.09 (0.61–1.93)	0.89 (0.38–2.09)	0.6 (0.23–1.57)	1.11 (0.54–2.28)	**Gefitinib (69%)**							
0.25 (0.04–1.62)	0.21 (0.03–1.52)	0.14 (0.03–0.63)	0.26 (0.04–1.64)	0.23 (0.0–1.4)	**Icotinib (97%)**						
**7.93 (2.48–25.38)**	**6.5 (1.5–28.11)**	**4.34 (1.04–18.11)**	**8.06 (3.11–20.85)**	**7.28 (2.21–24.02)**	**31.44 (3.9–253.3)**	**Erlotinib+ Bevacizumab (12%)**					
2.29 (0.93–5.65)	1.88 (0.53–6.67)	1.25 (0.37–4.27)	**2.33 (1.28–4.26)**	2.11 (0.82–5.39)	**9.09 (1.59–64.12)**	**0.29 (0.09–0.89)**	**Ramucirumab+ Erlotinib (38%)**				
6.67 (0.7–63.4)	5.46 (0.53–56.61)	3.65 (0.34–39.54)	6.77 (0.68–67.22)	**6.12 (1.09–54.08)**	**26.44 (1.56–446.81)**	0.84 (0.07–10.08)	2.91 (0.27–31.19)	**Gefitinib+ PbCT (17%)**			
**9.51 (3.38–27.78)**	**7.79 (2.32–26.13)**	**5.20 (1.43–18.99)**	**9.66 (3.14–29.73)**	**8.73 (3.69–16.98)**	**37.69 (5.11–278.1)**	1.20 (0.28–5.23)	**4.15 (1.16–14.85)**	1.43 (0.14–14.85)	**Apatinib + Gefitinib (9%)**		
1.77 (0.36–8.74)	1.45 (0.25–8.35)	0.97 (0.30–3.17)	1.8 (0.37–8.87)	1.63 (0.35–7.51)	**7.03 (2.71–18.25)**	0.22 (0.03–1.43)	0.77 (0.14–4.25)	0.27 (0.20–3.81)	0.19 (0.33–1.08	**PbCT (51%)**	
**4.91 (2.48–9.75)**	**4.02 (1.49–10.86)**	**2.69 (1.19–6.09)**	**4.99 (2.52–9.89)**	**4.51 (2.7–7.54)**	**19.48 (3.46–109.62)**	0.62 (0.19–2.0)	2.14 (0.86–5.33)	0.74 (0.08–6.91)	0.52 (0.19–1.41)	2.77 (0.66–11.7)	**PfCT (22%)**

Figure S8. Pooled estimates of the network meta-analysis after including only phase III trials in the second sensitivity analysis. (**A**) Pooled hazard ratios (95% confidence intervals) for overall survival (upper triangle) and progression-free survival (lower triangle). P-scores for overall survival (left) and progression-free survival (right) are indicated under each treatment. (**B**) Pooled odds ratios (95% confidence intervals) for adverse events of grade ≥ 3. P-scores are indicated under each treatment. Data in each cell are hazard or odds ratios (95% confidence intervals) for the comparison of row-defining treatment versus column-defining treatment. Hazard ratios or odds ratio less than 1 favor row-defining treatment. Significant results are in bold. NA, not available; PbCT, pemetrexed-based chemotherapy; PfCT, pemetrexed-free chemotherapy.

A

Progression-free survival

**Osimertinib (NA)**	NA	NA	NA	NA	NA	NA	NA	NA	NA	NA	NA
	**Dacomitinib (70%)**	0.76 (0.41–1.38)	1.05 (0.56–1.95)	**0.51 (0.37–0.71)**	**0.28 (0.12–0.67)**	1.93 (0.93–4.08)	1.60 (0.79–3.24)	0.82 (0.43–1.56)	0.77 (0.45–1.32)	**0.19 (0.10–0.38)**	**0.17 (0.11–0.26)**
		**Afatinib (51%)**	1.38 (0.72–2.65)	**0.68 (0.41–0.88)**	**0.37 (0.19–0.74)**	**2.57 (1.19–5.52)**	**2.11 (1.01–4.39)**	1.13 (0.63–2.03)	1.01 (0.52–1.99)	**0.25 (0.16–0.39)**	**0.23 (0.14–0.26)**
			**Erlotinib (69%)**	**0.49 (0.29–0.84)**	0.27 (0.11–0.67)	**1.96 (1.24–2.77)**	**1.52 (1.09–2.13)**	0.82 (0.43–1.56)	0.73 (0.37–1.77)	**0.18 (0.09–0.38)**	**0.16 (0.10–0.26)**
				**Gefitinib (30%)**	0.55 (0.25–1.22)	**3.78 (1.93–23.38)**	**3.10 (1.65–5.84)**	**1.67 (1.14–2.44)**	1.49 (0.96–2.32)	**0.37 (0.25–1.26)**	**0.33 (0.25–0.44)**
					**Icotinib (19%)**	**6.68 (2.55–18.81)**	**5.63 (2.12–14.74)**	**3.02 (1.34–6.80)**	**2.71 (1.09–6.67)**	0.67 (0.40–1.30)	0.60 (0.28–1.31)
						**Erlotinib+ Bevacizumab (97%)**	0.82 (0.49–1.39)	**0.44 (0.21–0.94)**	**0.39 (0.18–0.88)**	**0.10 (0.04–0.47)**	**0.09 (0.05–0.16)**
							**Ramucirumab+ Erlotinib (90%)**	0.54 (0.26–1.11)	0.48 (0.22–1.48)	**0.12 (0.05–0.47)**	**0.11 (0.06–0.19)**
								**Gefitinib+ PbCT (59%)**	0.90 (0.50–1.60)	**0.22 (0.12–0.41)**	**0.20 (0.13–0.31)**
									**Apatinib+ Gefitinib (52%)**	**0.25 (0.12–0.82)**	**0.22 (0.13–0.37)**
										**PbCT (7%)**	0.90 (0.50–1.60)
											**PfCT (5%)**

B

Adverse events of grade 3 or above

**Osimertinib (NA)**											
NA	**Dacomitinib (60%)**										
NA	1.36 (0.66–2.81)	**Afatinib (44%)**									
NA	0.46 (0.19–1.09)	**0.33 (0.15–0.75)**	**Erlotinib (89%)**								
NA	0.89 (0.53–1.49)	0.65 (0.39–1.09)	1.95 (0.97–3.94)	**Gefitinib (68%)**							
NA	**0.23 (0.06–0.87)**	**0.17 (0.06–.53)**	0.51 (0.12–2.02)	**0.26 (0.08–0.88)**	**Icotinib (98%)**						
NA	**3.75 (1.35–10.44)**	**2.75 (1.05–7.24)**	**8.22 (4.81–14.07)**	**4.21 (1.74–10.17)**	**16.05 (3.68–69.9)**	**Erlotinib+ Bevacizumab (9%)**					
NA	1.08 (0.42–2.76)	0.79 (0.33–1.90)	**2.36 (1.65–3.37)**	1.21 (0.55–2.65)	**4.6 (1.12–18.96)**	**0.29 (0.15–0.55)**	**Ramuciruma+ Erlotinib (56%)**				
NA	2.05 (0.48–8.66)	1.4 (0.40–5.66)	**4.48 (1.0–0.1)**	2.29 (0.6–8.82)	**8.75 (2.22–34.52)**	0.55 (0.11–2.68)	1.9 (0.41–8.88)	**Gefitinib+ PbCT (30%)**			
NA	**7.79 (3.70–16.40)**	**5.71 (2.73–11.95)**	**17.07 (7.05–41.29)**	**8.73 (5.10–14.94)**	**33.3 (8.83–125.56)**	2.08 (0.74–5.84)	**7.24 (2.79–18.78)**	3.81 (0.89–16.24)	**Apatini+ Gefitinib (1%)**		
NA	1.64 (0.53–5.10)	1.21 (0.49–3.0)	**3.60 (1.09–11.87)**	1.84 (0.67–5.05)	**7.03 (3.58–13.8)**	0.44 (0.12–1.62)	1.53 (0.44–5.31)	0.80 (0.24–2.66)	**0.21 (0.07–0.66)**	**PbCT (44%)**	
NA	**3.49 (1.99–6.13)**	**2.56 (1.62–4.04)**	**7.65 (3.94–14.85)**	**3.91 (3.11–4.91)**	**14.92 (4.5–19.49)**	0.93 (0.4–2.18)	**3.24 (1.53–6.89)**	1.71 (0.44–6.54)	**0.45 (0.25–0.80)**	2.12 (0.79–5.72)	**PfCT (19%)**

Figure S9. Pooled estimates of the network meta-analysis after excluding FLAURA studies in the third sensitivity analysis. (**A**) Pooled hazard ratios (95% confidence intervals) for progression-free survival. (**B**) Pooled odds ratios (95% confidence intervals) for adverse events of grade ≥3. P-scores are indicated under each treatment. Data in each cell are hazard or odds ratios (95% confidence intervals) for the comparison of row-defining treatment versus column-defining treatment. Hazard ratios or odds ratio less than 1 favor row-defining treatment. Significant results are in bold. NA, not available; PbCT, pemetrexed-based chemotherapy; PfCT, pemetrexed-free chemotherapy.
